# Five new genera of the subfamily Cylapinae (Insecta, Heteroptera, Miridae) from Australia

**DOI:** 10.3897/zookeys.1012.57172

**Published:** 2021-01-26

**Authors:** Anna A. Namyatova, Gerasimos Cassis

**Affiliations:** 1 St Petersburg State University, Faculty of Biology, Universitetskaya nab. 7/9, St. Petersburg, Russia St Petersburg State University St. Petersburg Russia; 2 University of Tyumen, Volodarskogo ul. 6, Tyumen, Russia University of Tyumen St. Petersburg Russia; 3 Zoological Institute, Russian Academy of Sciences, Universitetskaya nab. 1, St Petersburg, Russia Zoological Institute, Russian Academy of Sciences St. Petersburg Russia; 4 University of New South Wales, Evolution and Ecology Research Centre, School of Biological, Earth and Environmental Sciences, Randwick, Sydney, Australia University of New South Wales Sydney Australia

**Keywords:** Australian fauna, description, morphology, new species, plant bugs, taxonomy

## Abstract

Cylapinae is one of the poorly studied groups within the megadiverse family Miridae (Insecta: Heteroptera). In this paper, five monotypic genera from Australia are described as new to science. Two of those taxa, *Dariella rubrocuneata***gen. nov.** and **sp. nov.**, and *Labriella fusca***gen. nov.** and **sp. nov.** are assigned to the tribe Cylapini. Three taxa, *Callitropisca florentine***gen. nov.** and **sp. nov.**, *Laetifulvius morganensis***gen. nov.** and **sp. nov.** and *Micanitropis seisia***gen. nov.** and **sp. nov.** are placed into the tribe Fulviini. Habitus images, SEMs of external characters, illustrations of male and female genitalia, and distribution maps are provided for each species where possible. The systematic position and possible relationships of the newly described taxa are discussed.

## Introduction

Miridae is one of the largest hemimetabolous insect families, currently comprising more than 11,000 species and with numerous taxa yet to be described. Cylapinae is among the smallest subfamilies within the Miridae with approximately 100 genera and 500 species. Currently this subfamily includes the followings tribes: Bothriomirini, Fulviini, Cylapini, Rhinomirini, and Vanniini. Additionally, the subfamily Psallopinae is sometimes considered within Cylapinae as a tribe (Wolski and Henry 2015). These groups are very different morphologically and do not share any characters in common, which casts doubts on Cylapinae monophyly. The largest tribes, Fulviini and Cylapini, also seem to be groups of convenience and their generic composition will likely be revised in the future.

Most representatives of Cylapinae are distributed in tropical and subtropical regions. It is very likely the true diversity of this subfamily is still not comprehensive, as many of its representatives live in litter and under bark in tropical forests and cannot be collected using the most common mirid collection technique, plant sweeping and beating. During the last decade numerous Cylapinae taxa collected by hands, malaise traps, light traps, fogging, and bark spraying have been described from different regions (e.g., Carpintero and Cherot 2014; Wolski 2014, 2017; Wolski and Gorczyca 2014a; Wolski et al. 2016, 2017, 2018; Namyatova and Cassis 2019a; Tyts et al. 2020).

The Australian fauna of Cylapinae seems to be very little known, and currently it includes just 21 genera and 43 species from all tribes, except for Rhinomirini (Cassis and Gross 1995; Wolski and Gorczyca 2014b; Namyatova and Cassis 2016a, 2019a; Namyatova et al. 2019) and also four Psallopinae species (Namyatova and Cassis 2019b). It is estimated from museum collections, that Australian Cylapinae species diversity may reach at least 100 (Namyatova and Cassis 2016a). In this paper, we aim to further expand our knowledge on Australian cylapine fauna by describing five new monotypic Cylapinae genera within Cylapini and Fulviini and discussing their systematic position.

## Materials and methods

### Specimens

Eighty-two specimens were examined for this study. A unique specimen identifier (USI) was attached to each specimen, and collection event data were entered into the Arthropod Easy Capture Specimen Database (https://research.amnh.org/pbi/locality/index.php) and accessible through https://www.discoverlife.org/. The USI code starts with “UNSW_ENT” prefix for all the labels, except otherwise stated. The specimens are deposited in the following collections:

**AM**Australian Museum, Sydney, Australia;

**AMNH**American Museum of Natural History, New York, USA;

**NTM**Museum and Art Gallery of the Northern Territory, Darwin;

**QM**Queensland Museum, Brisbane, Australia;

**TMAG**Tasmanian Museums and Art Gallery, Hobart, Australia;

**SAMA**South Australian Museum, Adelaide, Australia;

**WAM**Western Australian Museum, Perth, Sydney.

### Dissection and terminology

The specimen dissection methodology follows Kerzhner and Konstantinov (1999). Terminology of male genitalia follows Kerzhner and Konstantinov (1999) and Konstantinov (2003), the aedeagus is described in repose. Terminology of female genitalia follows Davis (1955).

### Habitus and scanning electron micrograph images

The focus stacked habitus images were taken using Canon EOS 40D and Canon EOS 5D cameras, those stacks were concatenated using Helicon Focus ver. 6 software with standard setting. Scanning electron micrographs were made using a Hitachi TM-3000 tabletop electron microscope, the specimens were uncoated. The images were cropped and contrasted in Photoshop CS3 and CS5.1, the same software was used to create the figure plates.

### Measurements

Measurements have been completed using a Leica graticule and × 10 eyepieces, through a Leica MZ16 stereomicroscope. Measurements are provided in Table 1 in millimetres. The scale bars are 1 mm for habitus images and 0.1 mm for genitalia.

### Maps

The maps were completed using Simplemappr website (https://www.simplemappr.net/) (Shorthouse and Davis 2010) and processed with Photoshop CS5.1.

**Table 1.** Measurements.

Species		Length					Width		
		Body	Cun-Clyp	Pronotum	AntSeg 1	AntSeg2	Head	Pronotum	InterOcDi
*Dariella rubrocuneata*									
♂ (N = 5)	M1	2.06	1.48	0.40	0.25	0.67	0.54	0.73	0.21
	M2	2.23	1.50	0.42	0.25	0.71	0.54	0.77	0.23
	M3	2.19	1.54	0.42	0.27	0.65	0.50	0.73	0.19
	M4	2.25	1.56	0.44	0.31	0.77	0.52	0.77	0.21
	M5	2.08	1.46	0.44	0.25	0.67	0.52	0.75	0.21
*Labriella fusca*									
♂ (N = 5)	M1	2.96	2.10	0.48	0.25	0.88	0.69	0.94	0.23
	M2	2.88	2.04	0.48	0.25	0.79	0.65	0.92	0.21
	M3	2.81	1.98	0.44	0.25	0.83	0.65	0.90	0.23
	M4	2.67	1.94	0.46	0.25	0.75	0.65	0.85	0.23
	M5	2.81	2.00	0.46	0.25	0.77	0.65	0.85	0.21
♀ (N = 5)	F1	2.85	2.10	0.50	0.27	0.65	0.67	0.88	0.23
	F2	2.69	2.00	0.48	0.25	0.58	0.63	0.88	0.23
	F3	2.65	2.02	0.44	0.25	0.63	0.65	0.81	0.23
	F4	2.75	2.08	0.44	0.23	0.63	0.65	0.83	0.23
	F5	2.92	2.10	0.46	0.25	0.58	0.63	0.90	0.25
*Callitropisca florentine*									
♂ (N = 1)	M1	3.13	2.13	0.44	0.29	0.92	0.54	1.02	0.25
♀ (N = 2)	F1	3.00	2.25	0.46	0.21	0.83	0.54	1.00	0.27
	F2	3.04	2.27	0.42	0.21	0.77	0.56	0.92	0.29
*Laetifulvius morganensis*									
♂ (N = 2)	M1	3.10	1.98	0.42	0.25	0.77	0.60	0.94	0.21
	M2	3.04	2.04	0.46	0.25	0.77	0.60	0.92	0.23
*Micanitropis seisia*									
♂ (N = 5)	M1	3.21	2.33	0.54	0.35	0.98	0.56	1.15	0.23
	M2	–	–	0.58	0.33	1.00	0.63	1.13	0.25
	M3	3.38	2.35	0.50	0.33	0.94	0.60	1.08	0.23
	M4	3.19	2.42	0.52	0.31	0.94	0.60	1.17	0.23
	M5	3.13	2.29	0.54	0.29	–	0.58	1.10	0.21
♀ (N = 5)	F1	3.35	2.46	0.56	0.33	1.02	0.54	1.23	0.23
	F2	–	2.19	0.52	0.33	1.02	0.50	1.10	0.21
	F3	3.06	2.33	0.52	0.29	0.94	0.56	1.13	0.25
	F4	3.48	2.48	0.54	0.29	0.92	0.63	1.17	0.25
	F5	3.73	2.71	0.56	0.29	0.90	0.63	1.15	0.25

## Results

### Subfamily Cylapinae

#### Tribe Cylapini

##### Dariella gen. nov.

Taxon classificationAnimaliaHeteropteraMiridae

BE95FF97-D1FA-5232-88E0-B689FE15414B

http://zoobank.org/81EEFDFE-0ADE-4D5D-AC20-A4F3973EDCBB

###### Type species.

*Dariella rubrocuneata* sp. nov. by original designation.

###### Diagnosis.

**Figure 1.**
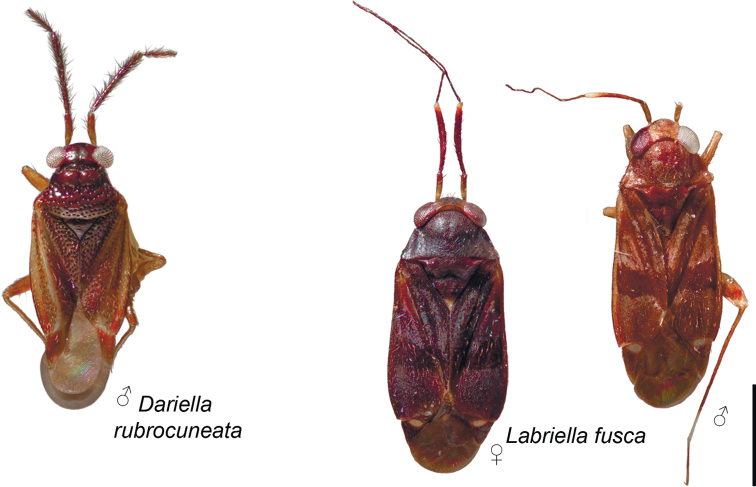
Habitus of *Dariella rubrocuneata* and *Labriella fusca*.

*Dariella* differs from other Cylapinae in the following combination of characters: macropterous; vertical head with antennal fossa placed above mandibular plate (Fig. 3A); elongate body, covered with short adpressed simple setae; pronotum, corium, and clavus deeply punctate (Fig. 3B, M); eye not pedunculated; vertex carinate posteriorly (Fig. 3B); base of pronotum wider than head; total antennal length shorter than body; antennal segment II slightly incrassate towards apex; antennal segments III and IV each shorter than segment II (Fig. 3E); buccula ring-like (Fig. 3A); apex of labium slightly surpassing posterior coxae; segments I and II not subdivided (Fig. 3D); collar delimited with deep groove (Fig. 3A, B); calli distinct with round shallow pit between them; scutellum flat (Fig. 3E, M); metathoracic scent gland evaporative area only slightly longer than wide with distinct vertical grove behind peritreme (Fig. 3F); outer margin of hemelytron slightly constricted anteriorly (Fig. 1); widest part of embolium subequal to 1/3 cuneus width at base; cuneus longer than wide at base (Fig. 3L); tarsal segment I shorter than segments II and III each (Fig. 3J); middle row of tiles on unguitractor reduced (Fig. 3K); parameres subequal in length and both with swelling in basal half directed outwards (Fig. 4D, F); phallotheca more extensively sclerotised apically, than basally; endosoma with single sclerotised area placed at right hand side (Fig. 4A, B).

###### Description.

**Male. Coloration** (Fig. 1). Head, pronotum, mesoscutum, scutellum mostly brown to dark brown; hemelytron, labium and appendages mostly pale brown to yellow with reddish tinge. For details see species description. **Surface and vestiture.** Dorsum shiny, without net-like pattern of microsculpture; posterior part of pronotum, clavus and corium with deep punctures (Fig. 3B, M); scutellum mostly smooth, serrate laterally (Fig. 3M); head, calli, embolium, cuneus and pleura smooth (Fig. 3B, F, L); dorsum and legs clothed with adpressed short simple setae, those setae on head and pronotum sparse; head with long suberect seta near inner margin of each eyes in dorsal view (Fig. 3B, C); antennae clothed with suberect setae mostly as long as or longer than antennal segment II width (Fig. 3E); anterior part of mesopleuron with area of dense short adpressed setae; posterior part of mesopleuron and metapleuron with sparse semi-adpressed setae (Fig. 3F). **Structure**. ***Head*.** Vertical, in dorsal view wider than long; eye not covering anterior margin of pronotum, not protruding; vertex carinate posteriorly (Fig. 3B); in anterior view head wider than high; antennal fossa attached near ventral margin of eye; clypeus separated from frons with shallow depression, its base placed slightly below ventral margin of eye (Fig. 3C); in lateral view head twice as high as long; eye slightly upraised above vertex, covering lateral margins of pronotum; distance between eye and ventral margin of head subequal to half of eye height; antennal fossa adjacent to eye, placed slightly above mandibular plate; mandibular and maxillary plates separated from head by shallow depression posteriorly; labrum triangular, shorter than labial segment I (Fig. 3A); buccula twice as long as high, ring-like, almost reaching posterior margin of head (Fig. 3A). ***Antenna*** (Fig. 3E). Shorter than body, segment I shorter than head width; segment II longer than head width, slightly incrassate apically; segment III subequal to half of segment II; segment IV ca. 1.5 × as long as segment III; segments I and II subequal in width and wider than segments III and IV each. ***Labium*** (Fig. 3D). Apex slightly surpassing hind coxa, its segments not subdivided; labial segment I surpassing base of forecoxa; labial segments I–III subequal in length; segment IV slightly shorter than segment III. ***Thorax*.** Pronotum wider than long, lateral margins concave in dorsal view, not carinate (Fig. 3A, B); collar delimited with deep sulcus, as wide as antennal segment I; posterior margin rounded and convex; calli swollen, occupying less than half of pronotum, separated from each other by depression and round pit (Fig. 3B); scutellum flat; mesoscutum almost entirely covered with pronotum (Fig. 3E, M); propleural apodeme mostly straight, its apical part inclined anteriorly and merging with collar sulcus; mesopleural apodeme oval; mesothoracic spiracle slit-like, without microsculpture around it; metathoracic scent gland evaporative area large, lateral margin reaching base of hind coxae, triangular, with distinct vertical groove; peritreme only slightly upraised, elongate; metepimeron subequal to 1/4–1/3 of mesopleuron in width (Fig. 3F). ***Hemelytron*.** Outer margin of hemelytron slightly constricted anteriorly (Fig. 1); ridge on clavus shallow, almost indistinct; claval commissure slightly more than twice longer than scutellum; medial fracture distinct, surpassing middle of corium (Fig. 3M); ridge along medial fracture absent; embolium wide, its widest part subequal to 1/3 of cuneus width at base; R+M more distinct basally; cuneus delimited with small incision, longer than wide at base; membrane with single cell, distance from cell apex to membrane apex subequal to cell length (Fig. 3L). ***Legs*.** Forecoxa length shorter than pronotum; coxae subequal in width and length; femora regular, not specifically widened; fore- and hind femora slightly wider than middle femur (Fig. 3G); segments II and III of hind tarsus subequal in length and each of them twice longer than segment I (Fig. 3J); claw with small subapical tooth; medial row of tiles on unguitractor reduced, having less tiles than lateral rows (Fig. 3K). ***Genitalia*.** See species description.

**Figure 2.**
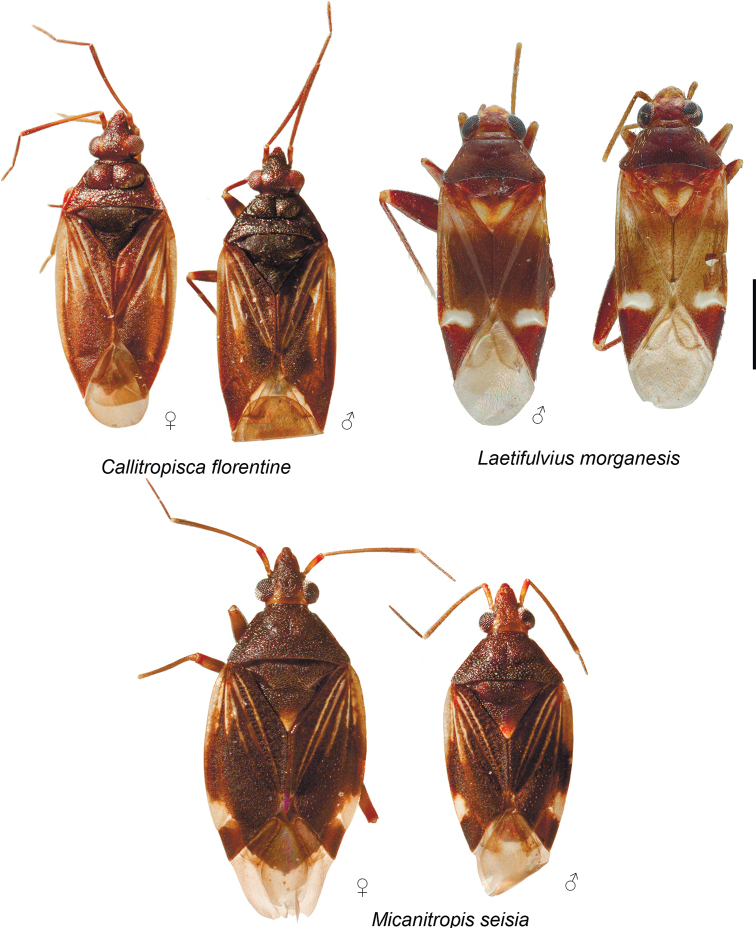
Habitus of *Callitropisca florentine*, *Laetifulvius morganensis*, and *Micanitropis seisia*.

**Female.** Unknown.

###### Etymology.

The genus is named after the sister of the first author (AN), Daria Namyatova. The gender is feminine.

###### Remarks.

**Figure 3.**
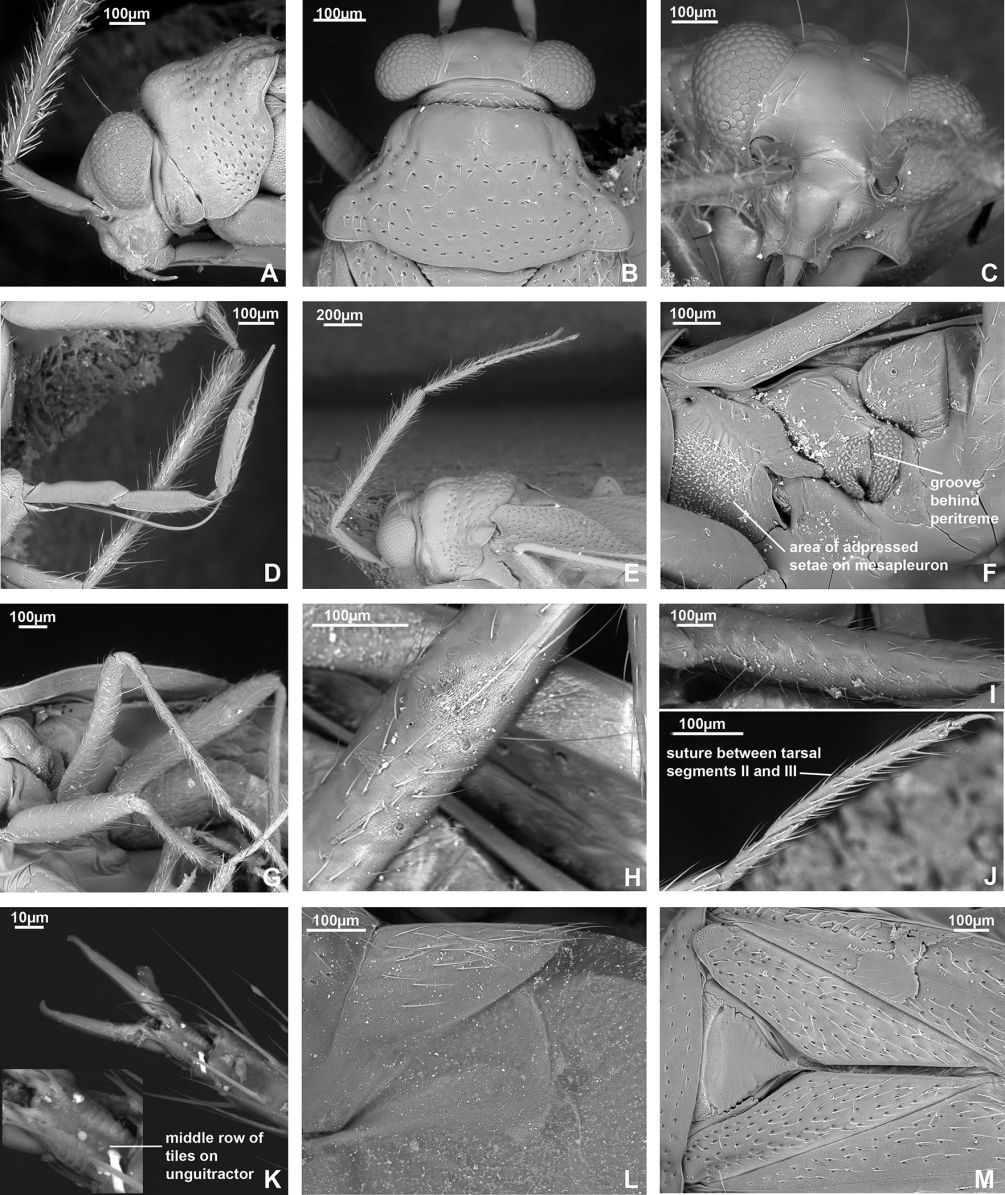
SEM images. *Dariella rubrocuneata***A** head and pronotum, lateral view **B** head and pronotum, dorsal view **C** head, anterior view **D** labium **E** antenna **F** pleura **G** legs **H** trichobothria on hind femur **I** trichobothria on middle femur **J** hind tarsus **K** pretarsus, ventral view **L** cuneus and membrane cell **M** scutellum, clavus and corium.

According to the present classification (Gorczyca 2000), *Dariella* does not belong to any Cylapinae tribe. It is similar to Cylapini and Bothriomirini in having punctate body (Fig. 3B, M), vertical head with antennal fossa placed above mandibular plate (Fig. 3A), and not subdivided labial segments I and II (Fig. 3D). See also Wolski (2017) and Namyatova et al. (2019) for detailed diagnoses of Cylapini and Bothriomirini respectively. However, most Cylapini species have antennae as long as or longer than body and antennal segments III and IV each longer than segment II (Wolski 2017), whereas in *Dariella* the antennae are shorter than body, and antennal segment III is shorter than segment II. In Bothriomirini, the body is more or less oval and stout, the collar is not delimited or weakly delimited, the scutellum is punctate, the tarsal segments are subequal in length (Namyatova et al. 2019), whereas in *Dariella* the body is elongate (Fig. 1), the collar is delimited with the deep sulcus (Fig. 3B), the scutellum is impunctate (Fig. 3M), and the tarsal segment I is shorter than each of segments II and III (Fig. 3J). Currently, we place *Dariella* in Cylapini, as it has affinities to some of its members, which also do not fully fit the diagnoses provided by Gorczyca (2000) and Wolski (2017).

**Figure 4.**
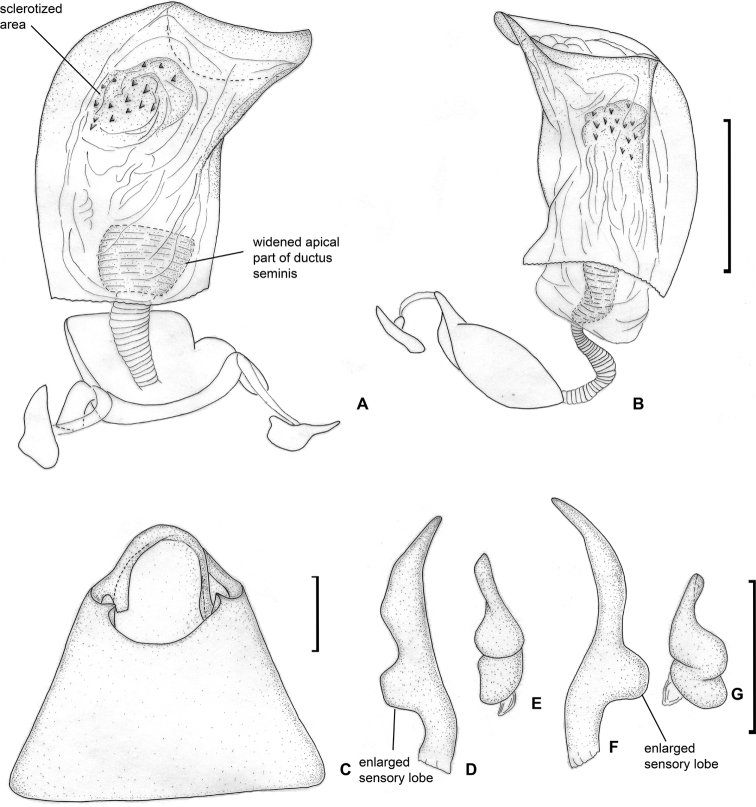
Male genitalia. *Dariella rubrocuneata***A** aedeagus, dorsal view **B** aedeagus, left lateral view **C** genital capsule, dorsal view **D** left paramere, dorsal view **E** left paramere, posterior view **F** right paramere, dorsal view **G** right paramere, posterior view.

*Dariella* is similar to the Neotropical genera *Corcovadocola* Carvalho, 1948 and *Cylapoides* Carvalho, 1952 as they also have short antennae (Carvalho 1948, 1952). Additionally, both those genera have a carinate vertex, apex of the labium reaches at least the hind coxa, and possess more or less developed calli. *Corcovadocola* and *Cylapoides* differ from *Dariella* in a convex scutellum and antennal segment III subequal in length to segment II. *Corcovadocola* additionally differs from *Dariella* in the lateral sides of pronotum being slightly carinate, and the narrower embolium. *Cylapoides* additionally differs in the eyes slightly pedunculate, the head width subequal to pronotum width and the cuneus as long as wide at the base (Carvalho 1952; Wolski 2017). *Dariella* is also similar to another Neotropical genus *Cylapinus*, as they both have a punctate body, wide embolium and pit between calli and very similar shape of parameres with the left and right parameres subequal in length and the basal half of both parameres with swelling directed outwards (Fig. 4B, F; Carvalho 1986: figs 12, 13). *Cylapinus* differs from the new genus in the body covered with erect setae and the cuneus being as long as wide (Carvalho 1986).

*Dariella* can be easily recognised externally from two Australian genera *Carvalhoma* Slater & Gross, 1977 and *Schizopteromiris*, currently placed in the Cylapini, as *Carvalhoma* has staphylinoid hemelytra and *Schizopteromiris* Schuh, 1986 has coleopteroid hemelytra in both sexes (Schuh 1986; Namyatova and Cassis 2016a). However, those two genera are similar to the new genus in having the antenna shorter than the body, a similar vertical head and punctate hemelytron. Additionally, *Carvalhoma* has a similar left paramere with the basal half having swelling directed outwards and the phallotheca sclerotised apically (Namyatova and Cassis 2016a), and most species of *Carvalhoma* also have the endosoma with the sclerite placed at right side, which is very similar to *Dariella* (cf. Fig. 4A, B; Namyatova and Cassis 2016a: figs 7A, B, 9A, B, 10A, B). Another character uniting *Carvalhoma*, *Schizopteromiris* and *Dariella* is the reduced middle row of tiles on the unguitractor (Fig. 3K; pers. obs. for *Schizopteromiris*; Namyatova and Cassis 2016a: fig. 2L). *Schizopteromiris* might be closely related to *Dariella*, as they both have paired setae near the inner margin of eye dorsally (Fig. 3B, C; pers. obs. for *Schizopteromiris*) and a very similar shape of the metathoracic scent gland evaporative area which is slightly longer than wide with distinct vertical grove behind peritreme (cf. Fig. 3F and Schuh 1986: fig 12).

##### Dariella rubrocuneata sp. nov.

Taxon classificationAnimaliaHeteropteraMiridae

6009AECD-6A2A-5F43-A8EF-C034C32BCAAC

http://zoobank.org/611929C3-2384-4614-A395-7D0CD9D4D369

Figs 1 , 3 , 4 , 15A

###### Material examined.

***Holotype*: Australia: Qld**: Mt Boolbun Sth (summit), 15.95°S, 145.1333°E, 950 m, 05 Nov 1995–11 Jan 1996, Monteith, Cook, Roberts, 1♂ (00043357) (QM). ***Paratypes*: Australia: Qld**: Graham Ra, 17.28333°S, 145.95°E, 550 m, 08 Dec 1995–09 Dec 1995, Monteith, Cook, Thompson, 1♂ (00043361) (QM). Koombooloomba Dam, Upper Tully, 17.8353°S, 145.605°E, 08 Dec 1989, Monteith, Thompson and Janetzki, 1♂ (00043362) (QM). Millaa Millaa Falls, 17.46667°S, 145.6°E, 800 m, 17 May 1995, G. B. Monteith, 1♂ (00045284) (QM). Mt Boolbun Sth (summit), 15.95°S, 145.1333°E, 950 m, 05 Nov 1995–11 Jan 1996, Monteith, Cook, Roberts, 3♂ (00043358, 00043360, 00043359) (QM).

###### Diagnosis.

Head, pronotum and pleura mostly brown to dark brown, corium and embolium yellow to pale brown with brown or reddish areas; antennal segment I yellow, reddish apically; segments II–IV mostly brown, segment IV whitish apically; labium yellow with reddish tinge; embolium reddish apically; cuneus red or pale brown with distinct reddish tinge (Fig. 1); endosoma with sclerotised area placed at right side and armed with small teeth (Fig. 4A, B).

###### Description.

**Male.** Body length 2.1–2.3. **Coloration** (Fig. 1). Head mostly brown to dark brown, sometimes with reddish tinge; antennal segment I yellow, reddish apically; segments II and III brown; segment IV brown, whitish apically; labium yellow with reddish tinge; pronotum, mesoscutum and scutellum brown to dark brown, often with reddish tinge; pleura mostly brown to dark brown; metathoracic scent gland evaporative area and metapleuron often reddish or with reddish tinge; clavus brown to reddish brown; corium and embolium yellow to pale brown with brown or reddish areas; embolium reddish apically; cuneus red or pale brown with distinct reddish tinge; membrane brown; legs mostly pale brown with red tinge; abdomen reddish brown. **Surface and vestiture.** See generic description. **Structure and measurements**. Body ca. 2.6–2.7 × as long as wide, ca. 2.8–3.0 × as long as pronotum width; head ca. 3.6–4.3 × as wide as long; vertex ca. 1.2–1.5 × as wide as eye; anterior view head ca. 1.3–1.4 × as wide as high; antennal segment I ca. 1.1–1.5 × as long as vertex, ca. 0.5–0.6 × as long as head width; segment II ca. 2.4–2.8 × as long as segment I, ca. 3.1–3.7 × as long as vertex, ca. 1.2–1.3 × as long as head width, ca. 0.9–1.0 × as long as pronotum width at base; segment III slightly shorter than segment I, and segment IV almost twice longer than segment III; pronotum ca. 1.7–1.9 × as wide as long, ca. 1.4–1.5 × as wide as head. ***Genitalia*.** Genital capsule triangular, without supragenital bridge (Fig. 4C); parameres subequal to each other in size and very similar in shape, slightly curved and with swelling in basal half directed outwards (Fig. 4D–G); phallotheca stronger sclerotised closer to apex than basally; ductus seminis short, its apical part widened and placed inside endosoma, somewhat sclerotised; endosoma not subdivided into vesica and conjunctiva, voluminous, with sclerotised area placed at right side and armed with small teeth (Fig. 4A, B).

**Female.** Unknown.

###### Distribution.

Known only from Australian Wet Tropics (Fig. 15A).

###### Collection methods.

The specimens were collected with flight intercept trap and pyrethrum knockdown.

###### Etymology.

The species is named for its red cuneus, *ruber* from Latin, meaning red.

##### Labriella gen. nov.

Taxon classificationAnimaliaHeteropteraMiridae

4BC4957C-81F3-523E-9EB3-F655956675C0

http://zoobank.org/6C2CE837-63F7-46DD-A270-76413C12572D

###### Type species.

*Labriella fusca* sp. nov. by original designation.

###### Diagnosis.

**Figure 5.**
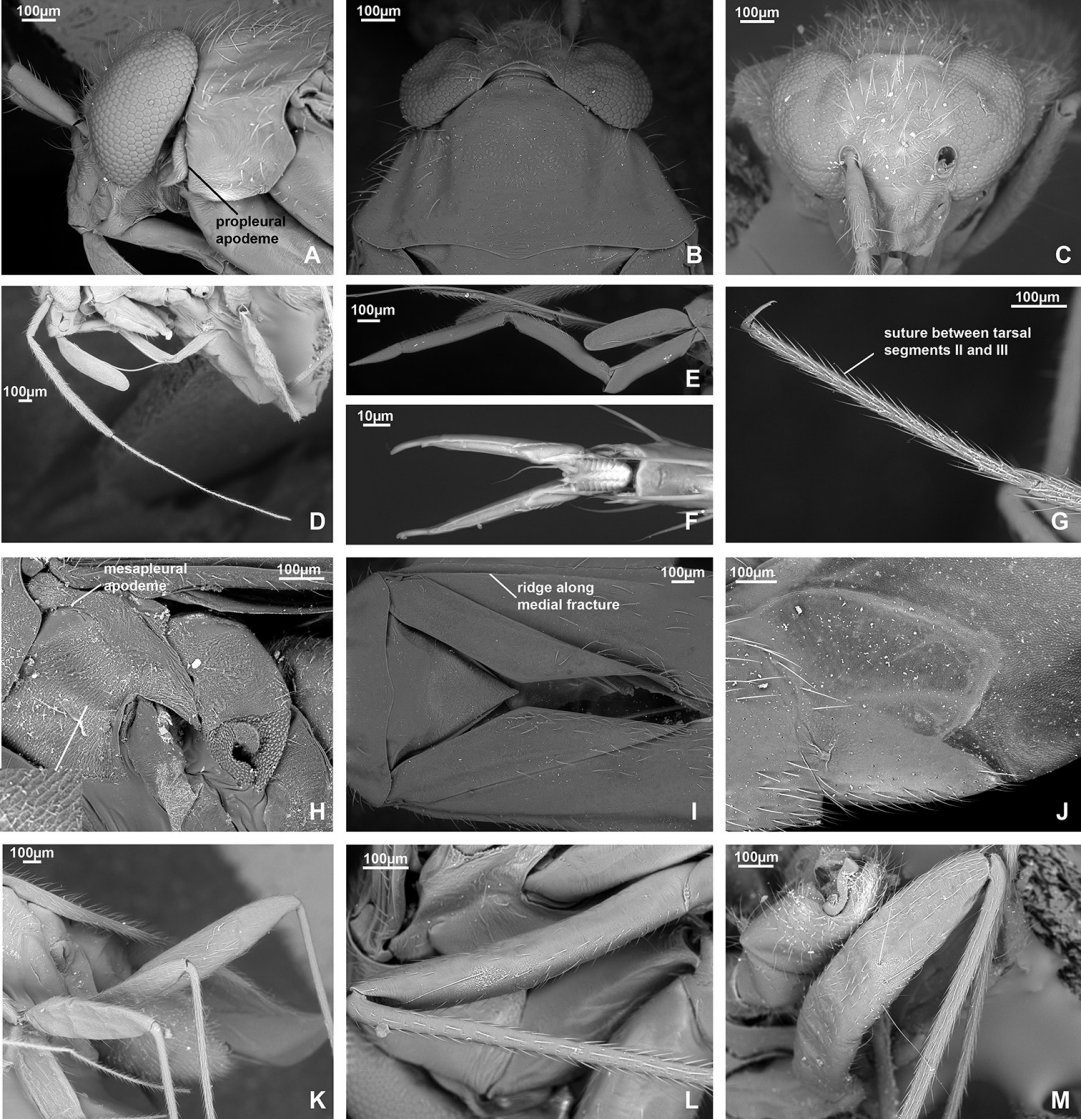
SEM images. *Labriella fusca***A** head and pronotum, lateral view **B** head and pronotum, dorsal view **C** head, anterior view **D** antenna **E** labium **F** pretarsus, ventral view **G** hind tarsus **H** pleura **I** scutellum, clavus and corium **J** cuneus and membrane cell **K** legs **L** trichobothria on middle femur **M** trichobothria on hind femur.

*Labriella* is distinguished from other Cylapinae by the following combination of characters: labrum longer than labial segment I, oval, flattened at sides (Fig. 5D, E); head vertical with antennal fossa located above mandibular plate (Fig. 5A); eye not pedunculate; vertex carinate, concave (Fig. 5B, C); eye covering anterior angle of pronotum (Fig. 5A, B); buccula not ring shaped, declivous posteriorly (Fig. 5A); total antenna length shorter than body with antennal segment II as thick as segment I, segment IV longest (Fig. 5D); apex of labium reaching abdominal segments IV–V; labial segments I and II not subdivided (Fig. 5E); collar very narrow, delimited with deep depression (Fig. 5B); mesepimeral apodeme slit-like; mesothoracic spiracle with microsculpture along anterior margin dorsally (Fig, 5H); corium with ridge along medial fracture (Fig. 5I); impunctate brown body, covered with semi-adpressed setae; pronotum and hemelytron not constricted; hemelytron not modified or shortened (Figs 1, 5I); femora not significantly enlarged (Fig. 5K); parempodia setiform (Fig. 5F).

###### Description.

**Male. Coloration** (Fig. 1). Mainly dark brown, for details see description of the species. **Surface and vestiture.** Dorsum and pleura glabrous, mostly matte, without punctation or rugosities (Fig. 5B, I, H); scutellum not serrate laterally (Fig, 5I); pleura with net-like pattern of microsculpture (Fig. 5H); body clothed with dark semi-adpressed setae, shorter than antennal segment II width, those setae shorter on appendages and almost absent on pleura (Fig. 5B, I, H). **Structure. *Head*.** In dorsal view head wider than long, vertical, vertex concave and carinate; eye covering anterior part of pronotum, not protruding (Fig. 5B); in anterior view head wider than high; antenna attached near ventral one third of eye, above ventral margins of eye; clypeus separated from frons by depression, its base placed slightly below antennal fossae, but above inferior margin of eye (Fig. 5C); in lateral view head ca. 1.5 × as high as long; eye slightly upraised above vertex, covering lateral margins of pronotum; distance from eye to ventral side of head subequal to third part of eye height laterally; eye covering anterior angles of pronotum; antennal fossa placed slightly above mandibular plate, adjacent to eye; mandibular and maxillary plate separated from head by distinct suture posteriorly; buccula twice as long as high, declivous posteriorly, not ring-like, almost reaching posterior margin of head (Fig. 5A); labrum as long as labial segment I, oval and flattened (Fig. 5D, E). ***Antenna*** (Fig. 5D). Total length shorter than body; segment I subequal to vertex width; segment II as wide as segment I, cylindrical and not incrassate apically; segment III and IV filiform, narrower than segments I and II; segment III subequal to half of segment II; segment IV ca. 2.5 × as long as segment III. ***Labium*** (Fig. 5D, E). Reaching abdominal segments IV–V, segments not subdivided; labial segment I slightly surpassing base of forecoxa; segments I, II and III subequal in length, segment IV subequal to half of segment III. ***Thorax*.** Pronotum wider than long; collar delimited, very narrow, narrower than antennal segment I (Fig. 5B); lateral margin of pronotum in dorsal view straight (Fig. 5B), in lateral view angulate, but not carinate (Fig. 5A); posterior margin of pronotum bisinuate (Fig. 5B); calli slightly upraised, occupying 2/3 of pronotum; calli separated with shallow depression between them; scutellum flat; mesoscutum exposed (Fig. 5I); propleural apodeme mostly straight, apical part inclined anteriorly (Fig. 5A), mesothoracic apodeme slit-like; mesothoracic spiracle open, slit-like, with small area of microsculpture along anterior margin dorsally; metathoracic gland evaporative area triangular, lateral margin reaching base of hind coxa; peritreme noticeably upraised, rounded, matte; metepimeron narrow (Fig. 5H). ***Hemelytron*.** Slightly narrowed anteriorly, and widened posteriorly; longitudinal ridge on clavus present, distinct; claval commissure almost twice longer than scutellum; medial fracture distinct, surpassing middle of corium; ridge along medial fracture present, surpassing middle of corium; embolium mostly narrow, apically widened, its width subequal to 1/6–1/7 of cuneus width at base (Fig. 1); R+M almost indistinct on posterior part of corium (Fig. 5I); cuneus delimited with pronounced incision, longer than wide; membrane with two cells (Fig. 5J). *Legs*. Forecoxa slightly longer than pronotum, slightly longer and as wide as middle and hind coxae; forefemur widened, approximately the same width as hind femur, each of them wider than middle femur (Fig. 5K); tarsus three-segmented, segment I and III subequal in length; segment II slightly longer than each of them; suture between segment II and III weak (Fig. 5G); claw with subapical tooth, middle row of tiles on unguitractor distinct, not reduced (Fig. 5F). ***Genitalia*.** See description for species.

**Female.** Similar to male, but antennal segment II wider and shorter. *Genitalia*. See species description.

###### Etymology.

The genus is named for its enlarged labrum. The gender is feminine.

###### Remarks.

*Labriella* cannot be confidently placed to any of the Cylapinae tribes based on the current classification and diagnoses (Gorczyca 2000). The combination of the vertical head, carinate vertex, antennae shorter than the body and the presence of the ridge along the medial fracture occurs in all representatives of Bothriomirini (Namyatova et al. 2019). However, bothriomirines are distinctly punctate, their labium not reaching the abdomen, they have a collar not delimited or shallowly delimited, the mesopleural apodeme round, and their mesothoracic spiracle without microsculpture. All those character states are absent in *Labriella* (see Diagnosis).

*Labriella* is similar to all Fulviini in that the total antennal length is shorter than the body, antennal segment II is as thick as segment I, and the labium is relatively long, the apex is reaching abdominal segments IV–V. However, *Labriella* differs from other Fulviini representatives in the possession of a vertical head (Fig. 5A), whereas in Fulviini it is mainly horizontal (Gorczyca 2000). Additionally, in all examined representatives of Fulviini the antennal fossa is located near the suture between the mandibular and maxillary plates (e.g., Figs 8F, 11C, 13I, see also Wolski 2010; Wolski et al. 2017, 2018; Namyatova and Cassis 2019a for more SEM images of Fulviini heads), whereas in *Labriella* it is located just above the mandibular plate (Fig. 5A).

*Labriella* fits many characters provided for the Cylapini diagnoses by Wolski (2017), e.g., the vertical head (Fig. 5B), labial segments I and II not subdivided (Fig. 5E), and the collar delimited with deep depression (Fig. 5B). However, some characters of *Labriella* do not fit the diagnoses. For example, in Cylapini, the anterior portion of the vertex is perpendicular to the rest of the vertex, the buccula is ring-like, the ventral margin of the eye barely reaches or does not reach the mandibular plate, and the antennae are thread-like. Whereas in *Labriella* the vertex is sloping more or less gradually, the eye reaches the maxillary plate, the buccula is not ring-like (Fig. 5A) and the antennae are not thread-like (Fig. 5D). We place *Labriella* into Cylapini based on the shared vertical head character. Based on the personal observations and the literature, all genera of Cylapini have this type of head, whereas the head is horizontal or sub-horizontal in all examined Fulviini and all other characters vary within both tribes.

Among species of Cylapini, *Corcovadocola* and *Cylapoides* Carvalho, 1952 are most similar to *Labriella* with the eyes being at least slightly covering the anterior angles of the pronotum and the antennal length being shorter than the body. *Cylapoides* differs from the new genus in the eyes being slightly pedunculate, the labium reaching the hind coxae and the body covered with erect setae (Carvalho 1952). *Corcovadocola* differs in having a brachypterous female, the vertex being only slightly concave and the lateral margins of pronotum being slightly emarginate (Carvalho 1948). The structure of the labrum was not included in the initial descriptions for all the above-mentioned genera (Poppius 1913; Carvalho 1948, 1952, 1986).

*Labriella* may be similar to the Neotropical genus *Tucuruisca* Carvalho, 1986 placed within the Fulviini and known only from the initial description. It also has a vertical head and the long labium, and its antennae are shorter than the body. According to the image, the eyes in *Tucuruisca* are also large, and are placed very close to or even slightly covering the anterior angles of the pronotum. *Tucuruisca* differs from *Labriella* in possessing a body covered with long erect hairs, the antennal segment IV being shorter than segment II and the thickened hind femora (Carvalho 1986).

##### Labriella fusca sp. nov.

Taxon classificationAnimaliaHeteropteraMiridae

67343D69-DC5D-5DE3-ABA9-E637AF483600

http://zoobank.org/A9574811-547D-4FEE-8878-92E250E6F481

**Figure 6.**
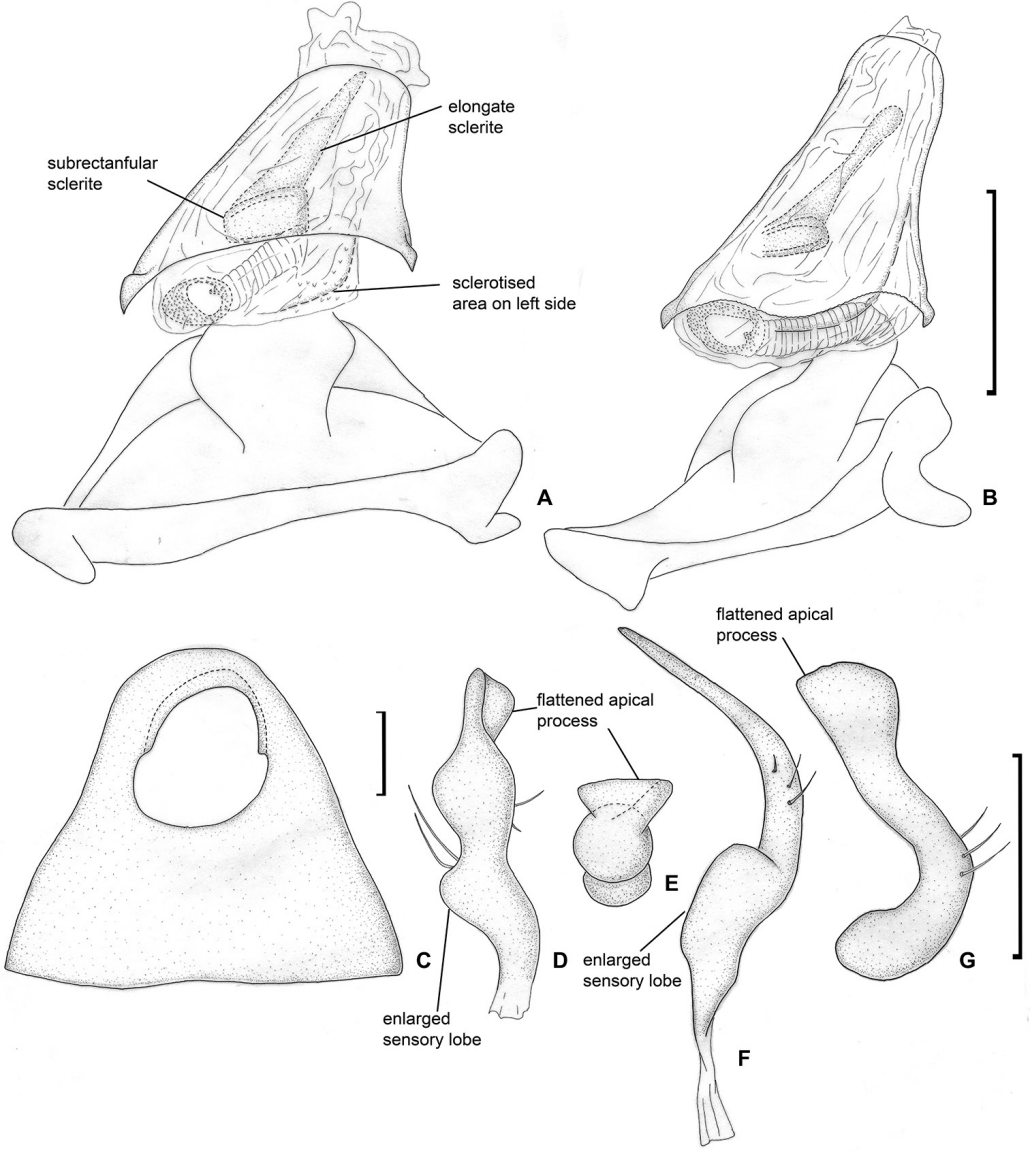
Male genitalia. *Labriella fusca***A** aedeagus, dorsal view **B** aedeagus, left lateral view **C** genital capsule, dorsal view **D** left paramere, dorsal view **E** left paramere, posterior view **F** right paramere, dorsal view **G** right paramere, posterior view.

**Figure 7.**
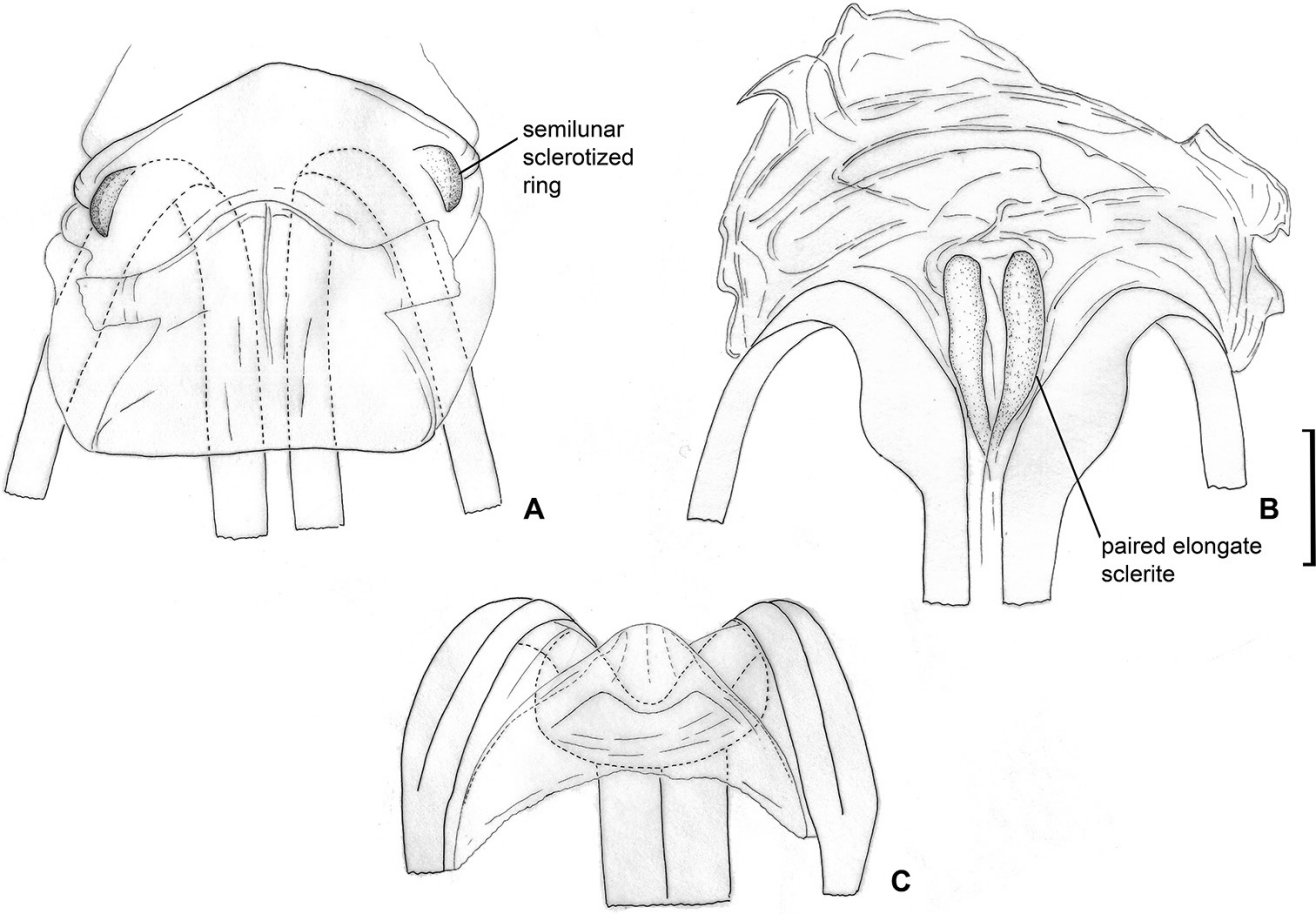
Female genitalia. *Labriella fusca***A** dorsal wall **B** ventral wall of bursa copulatrix **C** posterior wall of bursa copulatrix.

Figs 1 , 5 , 6 , 7 , 15B

###### Material examined.

***Holotype*: Australia: Qld**: Mossman Bluff Track, 5–10 km W Mossman, site 6, 16.46667°S, 145.3667°E, 860 m, 16 Dec 1988–30 Dec 1988, Monteith, Thompson and ANZSES, 1♂ (UNSW_ENT 00043296) (QM). ***Paratypes*: Australia: Qld**: 10 km SE El Arish, Laceys Creek nr Mission Beach, 17.86067°S, 146.08632°E, 40 m, 23 Jul 1982–05 Aug 1982, S. and J. Peck, 1♂ (00043320), 1 sex unknown (00043333) (AMNH). Bellenden Ker Range, 1 km S of Cable Tower 6, North Queensland, 17.23409°S, 145.86514°E, 500 m, 17 Oct 1981–05 Nov 1981, Earthwatch/QLD. Museum, 1♂ (00043312) (QM). Bellenden Ker Range, Cableway Base Stn, 17.271°S, 145.9°E, 100 m, 17 Oct 1981–09 Nov 1981, Earthwatch, 1 sex unknown (00043311) (QM). Cardwell Ra, Upper Broadwater Ck Valley, 18.3°S, 145.9333°E, 700 m, 17 Dec 1986–21 Dec 1986, Monteith, Thompson, Hamlet, 1♂ (00043317) (QM). Davies Ck Rd, 20 km SE Mareeba, 17.05°S, 145.6°E, 750 m, 04 Dec 1988–13 Dec 1988, Monteith and Thompson, 1♂ (00043319) (QM). Downey Ck, 25 km SE Millaa Millaa, 17.65°S, 145.7833°E, 400 m, 07 Dec 1988, G. Monteith and G. Thompson, 4♀ (00043291–00043294) (QM). Graham Ra, 17.28333°S, 145.9667°E, 550 m, 08 Dec 1995–09 Dec 1995, Monteith, Cook, Thompson, 3♀ (00043313, 00043314, 00043331) (QM). Hughes Rd, Topaz, 17.43333°S, 145.7°E, 650 m, 06 Dec 1993–25 Feb 1994, Monteith, Janetzki and Cook, 2♂ (00043309, 00043310) (QM). Lake Eacham, 17.28796°S, 145.62616°E, 750 m, 09 Dec 1989–14 Jan 1990, Monteith, Thompson and Janetzki, 1♂ (AMNH_PBI 00201880), 1 sex unknown (00043306) (QM). Mossman Bluff, 2 km ESE, 9 km W Mossman, 16.65°S, 145.5667°E, 1000 m, 17 Dec 1988–19 Dec 1988, Monteith and Thompson, 1♀ (AMNH_PBI 00404490) (QM). Mossman Bluff Track, 5–10 km W Mossman, N. Qld, Site 6, 16.46667°S, 145.3667°E, 860 m, 20 Dec 1989–15 Jan 1990, Monteith, Thompson and ANZSES, 1♂ (AMNH_PBI 00202018) (QM). Mossman Bluff Track,5–10 km W Mossman (Site1), 16.46667°S, 145.3667°E, 250 m, 20 Dec 1989–15 Jan 1990, Monteith, Thompson and ANZSES, 1♀ (AMNH_PBI 00404484), 1♂ (AMNH_PBI 00404486) (QM). Mossman Bluff Track, 5–10 km W Mossman (Site 5), 16.46667°S, 145.3667°E, 760 m, 16 Dec 1988–30 Dec 1988, Monteith, Thompson and ANZSES, 1♂ (00043297) (QM); 01 Jan 1989–16 Jan 1989, Monteith, Thompson and ANZSES, 1♂ (00043298) (QM); 20 Dec 1989–15 Jan 1990, Monteith, Thompson and ANZSES, 3♂ (00043117–00043119) (QM). Mossman Bluff Track, 5–10 km W Mossman, Site 7, 16.46667°S, 145.3667°E, 1000 m, 20 Dec 1989–15 Jan 1990, Monteith, Thompson and ANZSES, 3♂ (AMNH_PBI 00201868, AMNH_PBI 00201878, 00051481), 1♀ (AMNH_PBI 00404485) (QM). Mossman Bluff Track, 5–10 km W Mossman, site 6, 16.46667°S, 145.3667°E, 860 m, 16 Dec 1988–30 Dec 1988, Monteith, Thompson and ANZSES, 1♂ (00043295) (QM). Mossman Bluff Track, 9 km W Mossman, 16.44365°S, 145.29083°E, 1000 m, 17 Dec 1988, G. Monteith and G. Thompson, 1♂ (00043303) (QM). Mossman Bluff Track, 9 km W Mossman, Site 6, 16.44365°S, 145.29083°E, 860 m, 20 Dec 1989, Monteith and Thompson, 1♂ (00043302) (QM). Mossman Bluff Track, 10 km W Mossman, 16.45958°S, 145.27618°E, 1200 m, 17 Dec 1988, G. Monteith and G. Thompson, 1♂ (00043299), 2♀ (00043300, 00043301) (QM). Mt Finnigan summit, via Helenvale, 15.81667°S, 145.2833°E, 1100 m, 28 Nov 1985–30 Nov 1985, Monteith, Cook, Roberts, 1♂ (00043315) (QM). Mt Fisher, 7 km SW Millaa Millaa,Whiteing Rd, 17.55°S, 145.5667°E, 1200 m, 05 May 1983, G. B. Monteith, D. K. Yeates, 1♀ (AMNH_PBI 00404493) (QM). Mt Lewis Rd, 11 km from H’way (Site 1), 16.05°S, 145.2667°E, 1000 m, 18 Dec 1889–13 Jan 1990, Monteith, Thompson and ANZSES, 1♂ (00043304) (QM). Mt Lewis Rd,16 km from H’way (Site 2), 16.56667°S, 145.2667°E, 950 m, 18 Dec 1989–13 Jan 1990, Monteith, Thompson and ANZSES, 1♂ (00043305) (QM). Mt Spurgeon; 2 km SE, via Mt Carbine, 16.45°S, 145.2°E, 1100 m, 20 Dec 1988, Monteith and Thompson, 3♂ (00043120, 00043307, 00043308), 4♀ (00043121, AMNH_PBI 00404487-AMNH_PBI 00404489) (QM). Mt Williams 0.5 km NW, 16.91667°S, 145.6667°E, 870 m, 28 Nov 1997, G. B. Monteith, 1♀ (AMNH_PBI 00404491) (QM). PEI road. Topaz, 17.4°S, 145.68333°E, 580 m, 06 Dec 1993–25 Feb 1994, Monteith, Janetzki and Cook, 1♂ (00043316) (QM). Paluma Dam Rd, Site 2, 19.23333°S, 146.2167°E, 720 m, 17 Nov 1990–08 Dec 1990, Monteith and Seymour, 1♂ (00043318) (QM). Polly Ck, (Hasenpusch property), 17.46667°S, 146.0167°E, 50 m, 25 Nov 1994–10 Jan 1995, Monteith and Hasenpusch, 1 sex unknown (00043332) (QM). Russell River at Bellenden Ker Landing, 17.25°S, 145.94°E, 5 m, 24 Oct 1981–09 Nov 1981, Earthwatch, 1♂ (AMNH_PBI 00404492) (QM). Tully R. Xing, 10 km S Koombooloomba Dam, 17.9318°S, 145.61902°E, 750 m, 08 Dec 1989, Monteith, Thompson and Janetzki, 1♀ (00043330) (QM).

###### Diagnosis.

Body mostly brown to dark brown; clavus and corium with transversal stripe somewhat darker than rest of hemelytron (Fig. 1); labial segment III, scutellum apically, marking on cuneus at base, markings on abdomen, fore- and hind coxa at least basally, tibia apically, tarsi entirely or partly whitish yellow; endosoma with elongate sclerite, tapering apically; endosoma with sub-rectangular sclerite at base of elongate sclerite, and with sclerotised area on left side (Fig. 6A, B); dorsal labiate plate with semilunar sclerotised rings, those rings small, subequal to ca. 0.12–0.14 of dorsal labiate plate width (Fig. 7A).

###### Description.

**Male.** Body length 2.7–3.0. **Coloration** (Fig. 1). Mainly brown to dark brown, sometimes with reddish tinge; antennal segment II apically, labial segment III, scutellum apically, base of cuneus, markings on abdomen, fore- and hind coxae at least basally, tibia apically, tarsi entirely or partly whitish yellow. Antennal segment I also sometimes pale brown to whitish yellow. **Surface and vestiture.** As in generic description. **Structure and measurements.** Body ca. 2.6–2.8 × as long as wide, ca. 3.1–3.3 × as long as pronotum width. Head ca. 2.2–2.5 × as wide as long; vertex ca. 1.0–1.1 × as wide as eye; in anterior view head ca. 1.2–1.3 × as wide as high; antennal segment I ca. 1.1–1.2 × as long as vertex; segment II ca. 3.0–3.5 × as long as segment I, ca. 3.3–3.8 × as long as vertex width, 1.2–1.3 × as long as head width, ca. 0.9 × as long as pronotum width; pronotum ca. 1.9–2.1 × as wide as long, ca. 1.3–1.4 × as wide as head. ***Genitalia*.** Genital capsule simple, without supragenital bridge; apical part of ventral wall curved dorsally (Fig. 6C). Right paramere short, with swelling on basal half directed outwards; right paramere also with widened apical half; apical process of right paramere narrow in dorsal view and flat in posterior view (Fig. 6D, E); left paramere L-shaped, slightly longer than right paramere, mostly narrow with swollen basal part, swelling directed upwards and inwards; apical process of left paramere flat in posterior view (Fig. 6F, G), aedeagus with phallobase wider than phallotheca; endosoma not subdivided into vesica and conjunctiva; endosoma voluminous with sclerotised area on the left, elongate spicule tapering apically, and subrectangular spicule at base of elongate spicula; ductus seminis mostly membranous with coils, it curved right side within endosoma with secondary gonopore placed near right basal angle of phallotheca; ductus seminis with basal part sclerotised; ductus seminis around secondary gonopore oval, sclerotised, armed with microsculpture (Fig. 6A, B).

**Female.** Body length 2.7–2.9. **Coloration** (Fig. 1). As in male. **Surface and vestiture.** As in generic description. **Structure and measurements.** Body ca. 2.6–2.7 × as long as wide, ca. 3.1–3.3 × as long as pronotum width. Head ca. 1.9–2.2 × as wide as long; vertex ca. 1.0–1.3 × as wide as eye; in anterior view head ca. 1.1–1.2 × as wide as high; antennal segment I ca. 1.1–1.2 × as long as vertex; segment II ca. 2.3–2.7 × as long as segment I, ca. 2.3–2.8 × as long as vertex width, ca. 0.9–1.0 × as long as head width, ca. 0.7–0.8 × as long as pronotum width; pronotum ca. 1.8–2.0 × as wide as long, ca. 1.3–1.4 × as wide as head. ***Genitalia*.** Dorsal labiate plate with semilunar sclerotised rings, those rings small, ca. 0.12–0.14 × as wide as dorsal labiate plate width, and placed near posterior margin of dorsal labiate plate; lateral oviducts attached in the middle of dorsal labiate plate (Fig. 7A); ventral wall with paired elongate sclerites near vulva (Fig. 7B); posterior wall membranous, without any sclerotisation (Fig. 7C).

###### Distribution.

Known from numerous localities in the Australian Wet Tropics (Fig. 15B).

###### Collection methods.

The specimens of *Labriella fusca* were collected using pitfall traps, intercept traps, pyrethrum spraying of trees and logs and baited window trap.

###### Etymology.

Species is named so for its brown colour, *fuscus* from Latin meaning brown.

#### Tribe Fulviini

##### Callitropisca gen. nov.

Taxon classificationAnimaliaHeteropteraMiridae

E970162F-AA1F-53DB-BF72-83892F4FDAAC

http://zoobank.org/91DA3027-7449-4C4E-98FF-AEF2DA110A34

###### Type species.

*Callitropisca florentine* sp. nov. by original designation.

###### Diagnosis.

**Figure 8.**
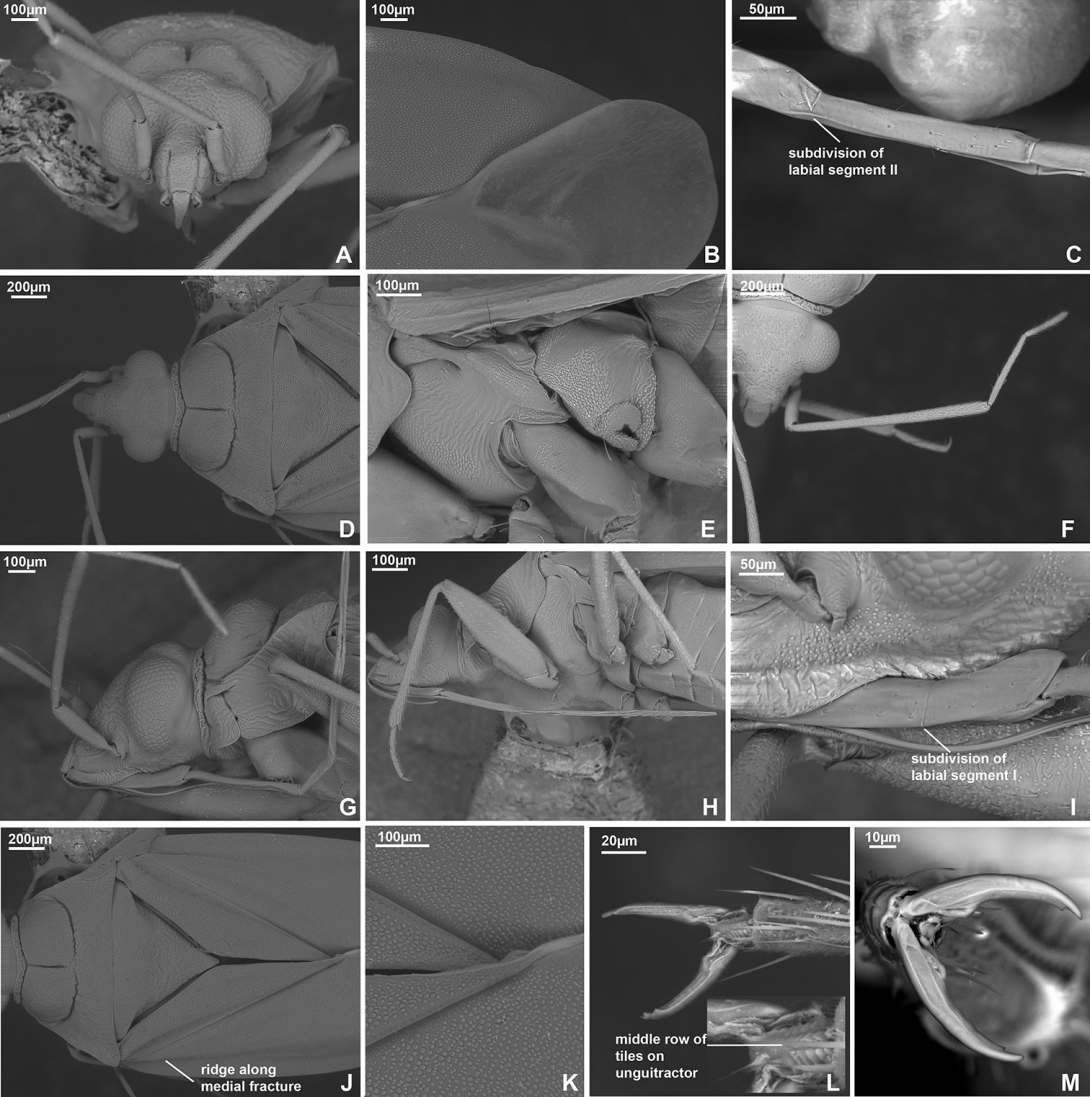
SEM images. *Callitropisca florentine***A** head, anterior view **B** cuneus and membrane cell **C** apical part of labial segment II **D** head and pronotum, dorsal view **E** pleura **F** antenna **G** head and pronotum, lateral view **H** labium **I** labial segment I **J** scutellum, clavus and corium **K** tubercles on hemelytron **L** pretarsus, ventral view **M** pretarsus, dorsal view.

**Figure 9.**
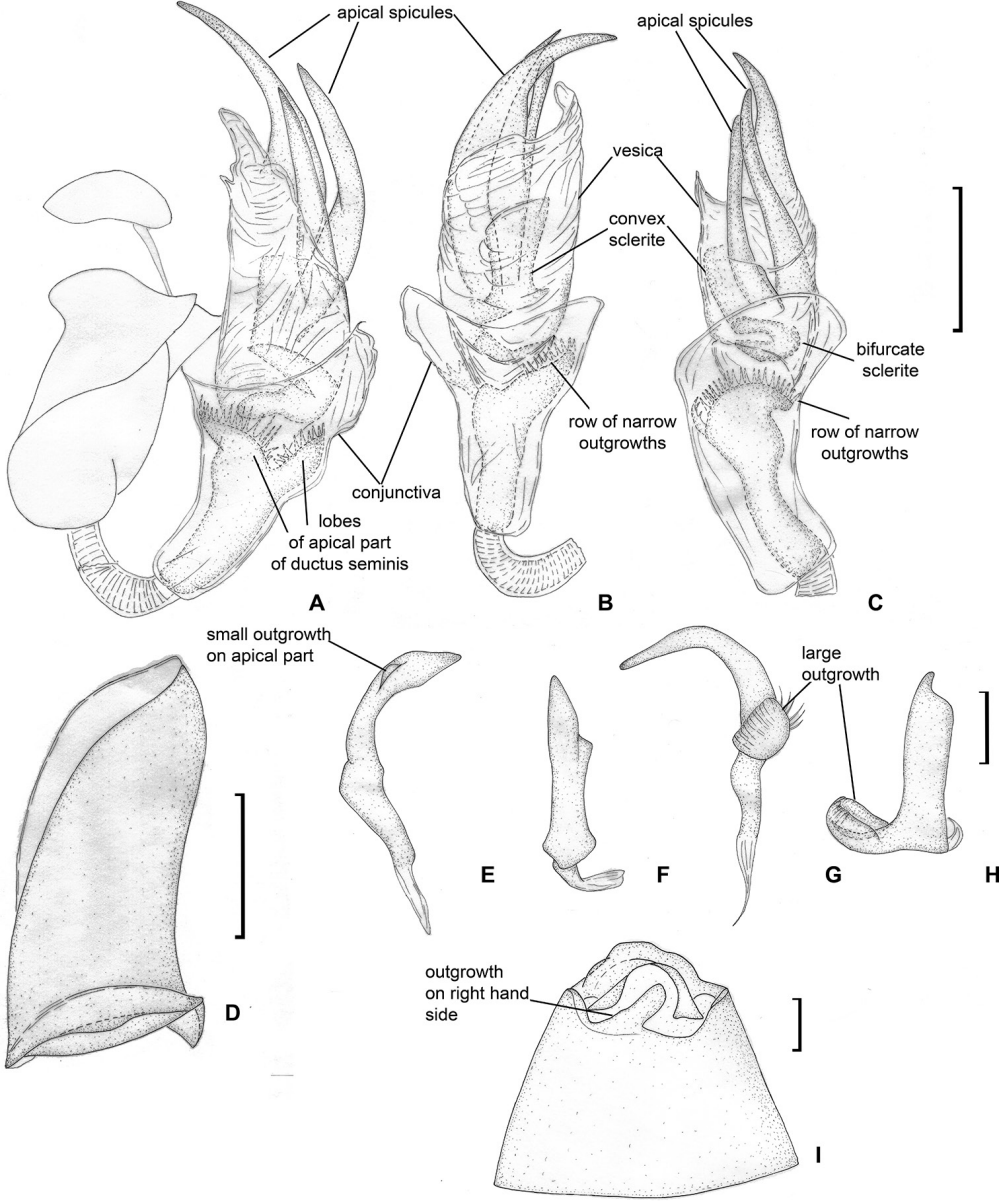
Male genitalia. *Callitropisca florentine***A** aedeagus, left lateral view **B** aedeagus, dorsal view **C** aedeagus, ventral view **D** theca **E** left paramere, dorsal view **F** left paramere, posterior view **G** right paramere, dorsal view **H** right paramere, posterior view **I** genital capsule.

**Figure 10.**
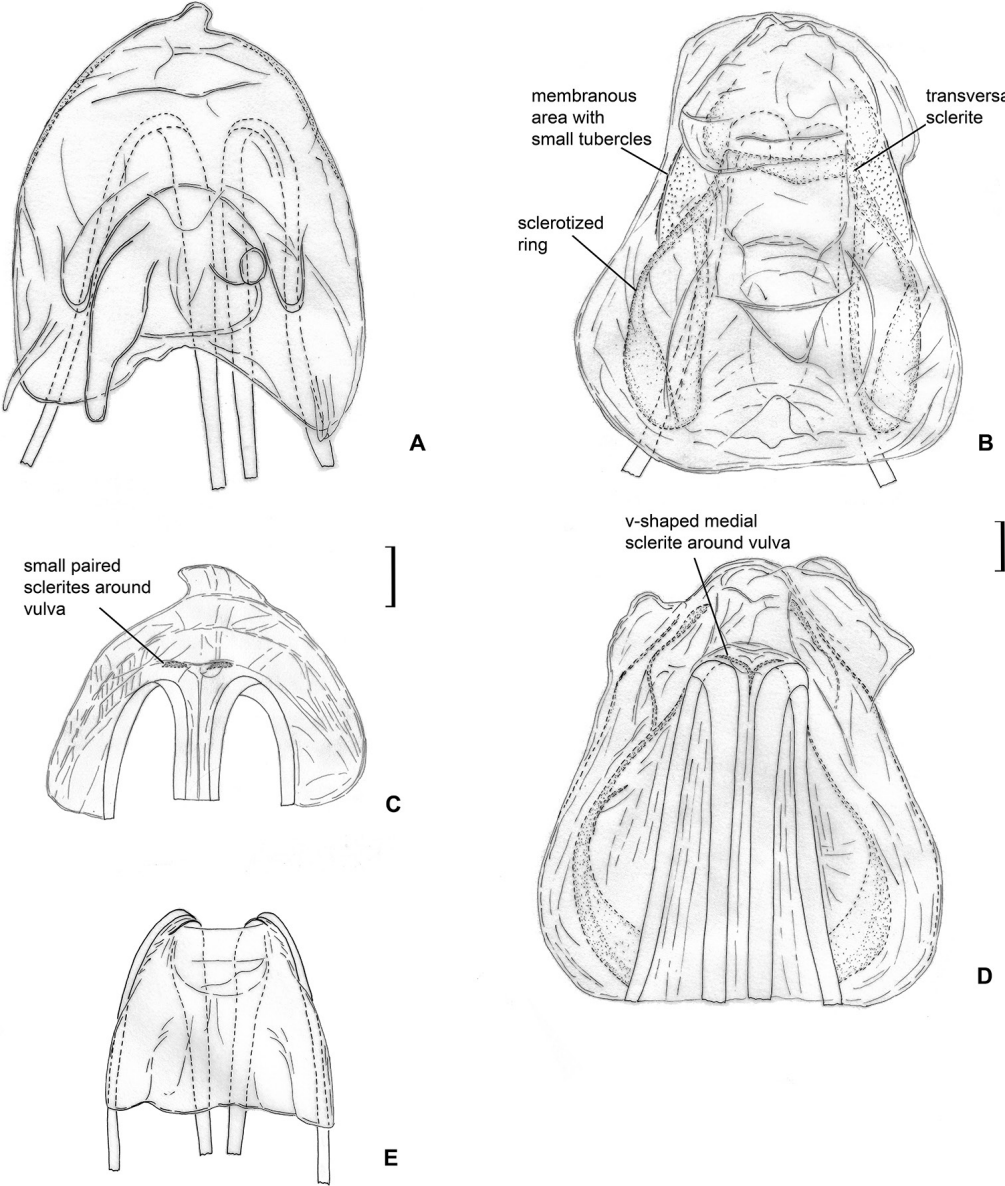
Female genitalia. *Callitropisca florentine***A** dorsal wall **C** ventral wall of bursa copulatrix **E** posterior wall of bursa copulatrix. *Micanitropis seisia***B** dorsal wall **D** ventral wall of bursa copulatrix.

*Callitropisca* can be recognised using following combination of characters: swollen and rounded calli, separated from each other; collar and rest of pronotum with distinct depression (Fig. 8D); vertex upraised above eye in lateral view (Fig. 8G); in lateral view distance between eye and ventral margin of eye equal to 1/6 of eye height (Fig. 8G); vertex not carinate (Fig. 8D, G); lateral margins of pronotum strongly carinate (Fig. 8G); apex of labial segment I not reaching pronotum (Fig. 8G); antennal segment I subequal to vertex width; antennal segment II straight, cylindrical, not widened or swollen (Fig. 8G, F); body impunctate, head and pronotum rugose; pleura smooth, without distinct rugosities (Fig. 8D, E, I–K); body covered with very short and sparse simple setae and small tubercles (Fig. 8K); hemelytron full, not shortened or modified, with whitish stripes on clavus and corium (Fig. 2); claval commissure twice as long as scutellum (Fig. 8J); metathoracic evaporative area large and triangular (Fig. 8E); left paramere with large outgrowth on basal part directed outwards and upwards (Fig. 9G, H), apical part of ductus seminis widened, with two lobes having row of narrow outgrowths along outer margin (Fig. 9A–C); dorsal labiate plate without sclerotised rings (Fig. 10A).

###### Description.

**Male. Coloration** (Fig. 2). Background colouration brown to dark brown, with yellow stripes on hemelytron. See species description for details. **Surface and vestiture.** Head and pronotum shiny, hemelytron matte (Fig. 2). Body impunctate; head and pronotum with distinct rugosities (Fig. 8D, G); pleura almost smooth, not visibly rugose (Fig. 8E); head laterally, scutellum and hemelytron clothed with small tubercles (Fig. 8G, J, K); net-like pattern of microsculpture present on mesopleuron ventrally (Fig. 8E); scutellum not serrate laterally (Fig. 8J). Body clothed with short adpressed setae, shorter than antennal segment II width; setae on dorsum very short and sparse (Fig. 8K); setae on antenna, pleura, legs and abdomen denser and longer; mesopleuron almost without setae; metapleuron with dense adpressed setae anteriorly (Fig. 8E); spines on tibiae short and pale; body impunctate (Fig. 8D, E, G, J, K). **Structure and measurements.** Body elongate. ***Head*.** Horizontal, dorsally as long as wide or slightly longer than wide, not carinate; eye not covering anterior margin of pronotum posteriorly, not protruding; vertex not carinate (Fig. 8D); in anterior view head wider than high; base of clypeus not delimited with depression, located above ventral margin of eye and antennal fossa; antennal fossa located near ventral margin of eye (Fig. 8A); in lateral view head slightly longer than high; vertex upraised above eye; base of clypeus not delimited with depression; distance between eye and ventral margin of head subequal to 1/6 of eye height; eye placed close to lateral margin of pronotum, but not covering it; antennal fossa removed from eye at distance equal to antennal fossa width, and located close to suture between mandibular and maxillary plates; mandibular and maxillary plates not separated by suture or depression from head; labrum triangular, shorter than labial segment I length; buccula elongate, 5–6 × as long as wide; distance between buccula and pronotum as long as buccula length (Fig. 8G). ***Antenna*.** Total length shorter than body; antennal segment I not widened, shorter than head width; antennal segment II cylindrical, slightly thinner than segment I, longer than head width; segment III slightly thinner than segment II, cylindrical, slightly shorter than segment II; segment IV subequal to half of segment III, and as thick as segment III (Fig. 8G, F). ***Labium*.** Apex reaching abdominal segments IV–V (Fig. 8H); labial segment I not surpassing posterior margin of head, subdivided in apical half (Fig. 8G, I); segment II almost twice as long as segment I, subdivided subapically, its apical part 9–10 × as long as wide (Fig. 8C, G); segment III subequal to 2/3 of segment II, more than 10 × as long as wide; segment IV subequal to 2/3 of segment III (Fig. 8H). ***Thorax*.** Pronotum wider than long; lateral margins straight in dorsal view, carinate; collar delimited dorsally and laterally (Fig. 8D, G); calli large, upraised, rounded, covering slightly more than half of pronotum, separated from each other and pronotum with distinct depression; posterior margin concave (Fig. 8D); scutellum flat, mesoscutum exposed, (Fig. 8J); propleural suture T-shaped (Fig. 8G); mesothoracic apodeme slit-like; mesothoracic spiracle oval, with one or two rows of microsculpture along anterior margin; metathoracic gland evaporative area large and triangular, lateral margin almost reaching base of hind coxa; peritreme upraised, rounded; metepimeron narrow (Fig. 8E). ***Hemelytron*.** Outer margin rounded (Fig. 2); ridge on clavus present, distinct; claval commissure twice longer than scutellum; medial fracture almost reaching middle of corium; ridge along medial fracture only basally visible; R+M basally visible and faint medially and apically (Fig. 8J); embolium wide, its widest part subequal to quarter of cuneus width at base (Fig. 2); cuneus delimited with faint suture, not incised; membrane with two cells; distance between cell and membrane longer than cell length (Fig. 8B). ***Legs*.** Forecoxa shorter than pronotum length, slightly wider and longer than middle and hind coxa; forefemur 4 × as long as wide, wider than and as long as middle femur (Fig. 8H); hind legs broken; claw with subapical tooth, unguitractor with medial row fully developed (Fig. 8L). ***Genitalia*.** See species description.

###### Etymology.

The genus is so named because of its swollen calli. The gender is feminine.

###### Remarks.

*Callitropisca* has all the diagnostic features for Fulviini, e.g., horizontal head, antenna shorter than body, forecoxae and forefemora enlarged, labium reaching middle of abdomen (Gorczyca 2000). It also has subdivided labial segments I and II, which is common for this group (Wolski and Henry 2015; Namyatova and Cassis 2019a). Therefore, we place *Callitropisca* into Fulviini. This genus differs from all other Fulviini representatives in the diagnostic characters, especially in possessing swollen and rounded calli, separated from each other, and the collar and rest of pronotum with a distinct depression (Fig. 8D).

*Callitropisca* is most similar, and, presumably, most closely related to *Micanitropis* and *Xenocylapidius*. *Callitropisca*, and *Micanitropis* have a similar colour pattern, with the body mainly brown to dark brown and whitish yellow longitudinal stripes on the clavus and corium (Fig. 2), the presence of rugosities on the head and pronotum (Figs 8A, D, G, J, 13A, E, I, M), the setae on the hemelytron sparse (Figs 8K, 13B), the presence of an outgrowth on the right side on posterior margin of genital capsule when viewed dorsally (Figs 9I, 14D), and the aedeagus with the ductus seminis having a row of narrow outgrowths along the apical margins (Figs 9A–C, 14A, B). The structure of the genital capsule for *Xenocylapidius* is unknown; however, it has a similar structure of the aedeagus (Wolski and Gorczyca 2014b). The authors named the structure “basal sac of endosoma”. However, it has similar shape and position as the apical part of the ductus seminis in *Callitropisca* and *Micanitropis*. In all those species, the left paramere has a large outgrowth in the basal half (Figs 9G, H, 14G, H; Wolski and Gorczyca 2014b: figs 19, 24, 29, 35, 40). The hemelytron of *Callitropisca* and *Micanitropis* is covered with rounded small tubercles (Figs 8K, 13B), and similar microstructure, although more elongate in shape, was also observed in the examined species of *Xenocylapidius* by the first author. Both, *Xenocylapidius* and *Micanitropis* differ from *Callitropisca* in the calli being less developed, and not surrounded by a distinct depression (Fig. 13E; Wolski and Gorczyca 2014b: figs 1–8), the frons not raised above the eye in lateral view (Fig. 13I; Wolski and Gorczyca 2014b: figs 9–15), and the labial segment I reaching the pronotum (Fig. 13I, N; Wolski and Gorczyca 2014b: figs 9–15). *Micanitropis* additionally differs from *Callitropisca* in the pleura being noticeably rugose (Fig. 13J), the claval commissure being only slightly longer than clavus (Fig. 13M) and the dorsal labiate plate possessing large sclerotised rings (Fig. 10B).

##### Callitropisca florentine sp. nov.

Taxon classificationAnimaliaHeteropteraMiridae

6137C146-AF11-59B6-A6D0-EB22180CC96F

http://zoobank.org/F6544F7C-8B3F-4742-9075-ECCDC55352A6

Figs 2 , 8 , 9 , 10A, C, E , 15A

###### Material examined.

***Holotype*: Australia: Tas**: 29 km WNW Maydena on Eleven Rd, Florentine Valley, 42.76667°S, 146.4°E, 460 m, 01 Feb 1980–06 Feb 1980, A. F. Newton, M. K. Thayer, 1♂ (UNSW_ENT 00043066) (TMAG). ***Paratypes*: Australia: Tas**: 29 km WNW Maydena on Eleven Rd, Florentine Valley, 42.76667°S, 146.4°E, 460 m, 01 Feb 1980–06 Feb 1980, A. F. Newton, M. K. Thayer, 1♀ (00043068) (AMNH). Strahan, 42.15°S, 145.3333°E, 52 m, Lea and Carter, 1♀ (00043067) (SAMA).

###### Diagnosis.

Characterised by head, pronotum, scutellum and pleura dark brown, and hemelytron brown; antennal segments mostly pale brown to brown; labial segment I red; hemelytron with whitish yellow stripes on clavus, along claval suture and on corium, and embolium whitish basally; coxa mostly reddish brown or reddish, fore- and middle femora mostly brown; foretibia pale brown, middle tibia reddish basally and yellow apically (Fig. 2); vesica with three sclerites elongate and acute apically and two sclerotised areas, one of those areas convex and placed on the left, and another one bifurcate, placed at base of spicules (Fig. 9A–C).

###### Description.

**Male.** Body length 3.1. **Coloration** (Fig. 2). ***Head*.** Dark brown with faint pale brown marking near inner margin of eye. Antennal segment I brown basally, yellow to pale brown medially and red apically; segments II–IV uniformly brown; labial segment I red, segments III and IV pale brown to brown. ***Thorax*.** Pronotum and scutellum dark brown to black; pleura dark brown. ***Hemelytron*.** Mostly brown with two whitish yellow stripes on clavus, whitish yellow stripe on claval suture and two stipes on corium; stripes not reaching or barely reaching middle of corium; embolium whitish yellow basally; membrane pale brown with brown cells. ***Legs*.** Coxae reddish brown, whitish yellow apically; fore- and middle femora brown with wide pale brown band in apical half and whitish yellow at extreme apex; foretibia pale brown; middle tibia reddish basally and yellow apically; tarsi pale brown. **Surface and vestiture.** See generic description. **Structure and measurements.** Body ca. 2.7 × as long as wide, ca. 3.1 × as long as pronotum width; head horizontal, as wide as long, in anterior view head ca. 1.4 × as long as high; antennal segment I ca. 1.2 × as long as vertex width, ca. 0.5 × as long as head width; antennal segment II ca. 3.1 × as long as segment I, ca. 3.7 × as long as vertex width, ca. 1.7 × as long as head width, ca. 0.9 × as long as pronotum width; pronotum ca. 1.9 × as wide as head, ca. 2.3 × as wide as long. ***Genitalia*.** Genital capsule as long as wide, with outgrowth on right hand side on posterior margin dorsally (Fig. 9I). Parameres r-shaped, subequal in length; basal part of right paramere with angulate swelling directed inwards and rounded outgrowth directed upwards; apical part of right paramere widened with small tubercle apically; left paramere with large outgrowth on basal part directed outwards and upwards, apical process narrow and elongate (Fig. 9E–H); theca without outgrowths (Fig. 9D); endosoma subdivided into vesica and conjunctiva; vesica with three elongate spicules acute apically, and with two sclerites, one of them convex and placed on the left, and another one bifurcate, placed at base of spicules; apical part of ductus seminis strongly sclerotised, secondary gonopore surrounded by two wide lobes with row of narrow outgrowths along outer margin (Fig. 9A–C).

**Female.** Body length 3.0. **Coloration** (Fig. 2). Head dark brown with yellow marking near inner margin of eye, or reddish brown with dark brown area around antennal fossa, yellow marking near inner margin of eye and paired markings on vertex medially closer to frons; antennal segment II changing colour gradually from pale brown to brown or from yellow to red; segment IV mostly brown, pale brown apically; pronotum brown to dark brown; pleura dark brown with reddish tinge or with red metapleuron including metathoracic scent gland evaporative area; coxae reddish brown or reddish, whitish yellow apically; legs as in male, but with more reddish tinge; abdomen reddish brown. **Surface and vestiture.** See generic description. **Structure and measurements.** Body ca. 2.5 × as long as wide, ca. 3.0–3.3 × as long as pronotum width; head horizontal, ca. 1.2 × as wide as long; in anterior view head ca. 1.4–1.5 × as long as high; antennal segment I ca. 0.7–0.8 × as long as vertex, ca. 0.3–0.4 × as long as head width; antennal segment II ca. 3.7–4.0 × as long as segment I, ca. 2.6–3.1 × as long as vertex, ca. 1.4–1.5 × as long as head width, ca. 0.8 × as long as pronotum width; pronotum ca. 1.6–1.9 × as wide as head, ca. 2.2 × as wide as long. ***Genitalia*.** Dorsal labiate plate without any sclerites; lateral oviducts placed in anterior half of dorsal labiate plate (Fig. 10A); ventral wall with small paired sclerites around vulva (Fig. 10C); posterior wall of bursa copulatrix without sclerites (Fig. 10E).

###### Distribution.

Known only from western Tasmania (Fig. 15A).

###### Collection techniques.

Unknown.

###### Etymology.

The species is named after Florentine valley, where two specimens of this species were collected.

##### Laetifulvius gen. nov.

Taxon classificationAnimaliaHeteropteraMiridae

79A88184-D9D1-557A-AEB9-456E0999E4C1

http://zoobank.org/0858F4B1-6362-4075-8AF9-31422D016EB1

###### Type species.

*Laetifulvius morganensis* sp. nov. by original designation.

###### Diagnosis.

**Figure 11.**
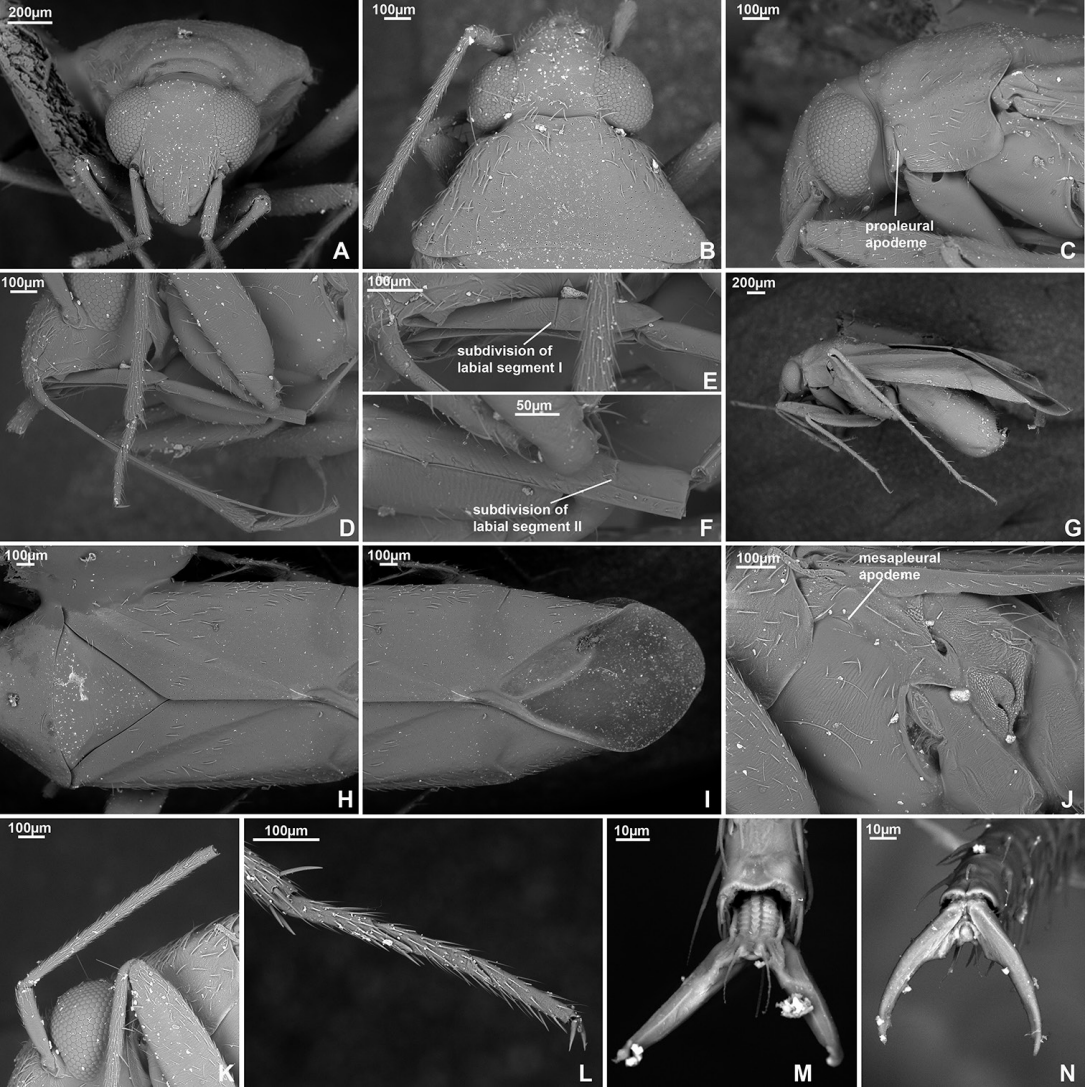
SEM images. *Laetifulvius morganensis***A** head, anterior view **B** head and pronotum, dorsal view **C** head, lateral view **D** labial segments I and II **E** labial segment I **F** apical part of labial segment II **G** legs **H** scutellum, clavus and corium **I** cuneus and membrane cell **J** pleura **K** antennal segments I and II **L** hind tarsus **M** pretarsus, ventral view **N** pretarsus, dorsal view.

**Figure 12.**
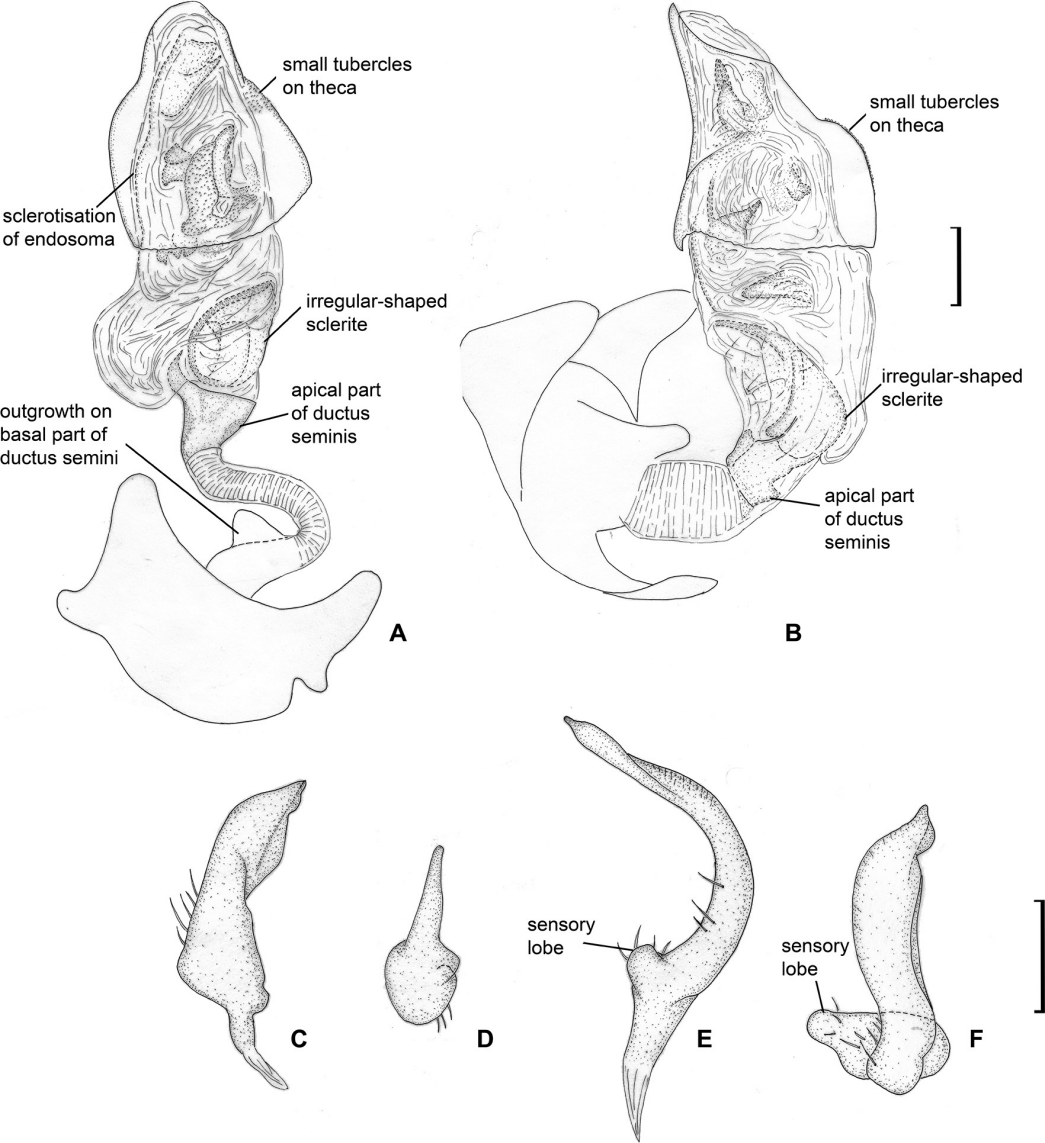
Male genitalia. *Laetifulvius morganensis***A** aedeagus, dorsal view **B** aedeagus, left lateral view **C** left paramere, dorsal view **D** left paramere, posterior view **E** right paramere, dorsal view **F** right paramere, posterior view.

Differs from other representatives of Cylapinae in the following combinations of characters: head semi-horizontal, antennal fossa attached near depression between mandibular and maxillary plates (Fig. 11C); antennal segment I length subequal to vertex width; eye located close to pronotum and slightly covering its anterior angle (Fig. 11B, C); vertex not carinate (Fig. 11B, C); labial segment I only slightly surpassing anterior margin of head, subdivided with suture (Fig. 11D, E), segment II subdivided with shallow suture apically (Fig. 11F); calli flat, indistinct (Fig. 6B); lateral margins of pronotum not carinate, rounded (Fig. 11C); collar delimited with shallow depression, relatively wide, wider than antennal segment I (Fig. 11B); evaporative area large and triangular (Fig. 11J); cuneus slightly longer than wide (Fig. 11I); forefemora not enlarged; hind femur twice wider than forefemur; tarsal segments subequal in length (Fig. 11L); body impunctate, clothed with simple semi-adpressed setae (Figs 2, 11B, H–J); endosoma not subdivided into vesica and conjunctiva (Fig. 12A, B).

###### Description.

**Male. Coloration** (Fig. 2). Mainly reddish brown with whitish yellow, yellow and pale brown markings. **Surface and vestiture** (Fig. 11B, C, H–J). Dorsum glabrous, shiny, impunctate and not rugose, without distinct tubercles or net-like pattern of microsculpture on dorsum and pleura; scutellum not serrated laterally; body clothed with pale sparse simple semi-adpressed setae, shorter than antennal segment II width, those setae denser on antennae and tibiae and very rare on pleura. **Structure. *Head*.** Semi-horizontal, in dorsal view head wider than long; vertex not carinate posteriorly; eye not protruding, covering anterior part of pronotum (Fig. 11B); in anterior view head slightly wider than high; antenna attached near ventral margin of eye; clypeus not separated from frons by depression, its base placed slightly above ventral margin of antennal fossae (Fig. 11A); in lateral view head as high as long; vertex not upraised above eye; eye removed from ventral side of head at distance subequal to fifth part of eye height, not covering anterior angles of pronotum; antennal fossa adjacent to eye, placed near suture between mandibular and maxillary plates, mandibular and maxillary plates not separated from head by suture posteriorly; labrum triangular, shorter than labial segment I; buccula elongate, ca. 5–6 × as long as high; distance between buccula and pronotum subequal to buccula length (Fig. 11C, D). ***Antenna*.** Segment I subequal to vertex width; segment II as wide as segment I, cylindrical, not incrassate apically (Fig. 11K). ***Labium*.** Labial segment I subdivided in apical half (Fig. 11E), apex reaching base of forecoxa (Fig. 11D); segment II slightly longer than segment I, subdivided apically with shallow suture (Fig. 11F). ***Thorax*.** Pronotum wider than long, lateral margins straight in dorsal view (Fig. 11B), in lateral view not carinate, rounded (Fig. 11C); collar delimited with shallow suture, relatively wide, wider than antennal segment I (Fig. 11B); posterior margin of pronotum concave (Fig. 11B, H); calli flat, almost indistinct, occupying less than half of pronotum (Fig. 11B); scutellum flat; mesoscutum exposed (Fig. 11H); propleural apodeme T-shaped (Fig. 11C); mesepimeral apodeme slit-like; mesepimeral spiracle oval, with wide area of microsculpture along anterior margin dorsally; metathoracic gland evaporative area triangular, lateral margin almost reaching base of hind coxa; peritreme upraised, rounded; metepimeron narrow (Fig. 11J). ***Hemelytron*** (Fig. 11H, I). Outer margin almost straight; clavus with longitudinal ridge; claval commissure longer than scutellum; medial fracture reaching middle of corium, but not surpassing it; ridge along medial fracture shallow, present basally and medially; white marking on corium posteriorly slightly upraised; R+M visible over entire length; embolium narrow, its widest part subequal to 1/5–1/6 of cuneus width; cuneus delimited, slightly longer than wide, its base not incised; membrane with singe cell, distance between cell and apex of membrane longer than cell length. ***Legs*.** Forecoxa as long as pronotum; fore- and hind coxae subequal in size, middle coxa slightly smaller than forecoxa; forefemur slightly longer than pronotum, ca. 4 × as long as wide, as long as and slightly wider than middle femur; hind femur twice as wide and ca. 1.5 × as long as forefemur (Fig. 11G); hind tarsus three-segmented, segments subequal in length (Fig. 11L); claw with subapical tooth, middle row of tiles on unguitractor full (Fig. 11M). ***Genitalia*.** See species description.

**Female.** Unknown.

###### Etymology.

The species is named for its colourful appearance, *laetus* from the Latin meaning colourful. The gender is masculine.

###### Remarks.

In Cylapinae tribe diagnoses, the length of the antennae and labium are among the most important characters (Gorczyca 2000; Cassis et al. 2003; Wolski 2017; Namyatova et al. 2019; Namyatova and Cassis 2019a), and *Laetifulvius* representatives do not match the diagnoses for these traits. However, they do have antennal segments I and II subequal in width, an impunctate body, labial segments I and II subdivided, antennal fossa placed near suture between mandibular and maxillary plates, and this combination of characters is typical for Fulviini. Therefore, we place *Laetifulvius* in this tribe. Although most Fulviini have a horizontal head, in some its genera it is also subhorizontal, e.g., *Mycetocylapus* Poppius, 1914 (Namyatova and Cassis 2019a), *Trynocoris* Herring, 1976 (Herring 1976), *Fulviella* Carvalho, 1991 (Carvalho 1991).

*Laetifulvius* may be related to *Phylocylapus* Poppius, 1913, as according to the initial description, the latter has a vertical head, wide collar, flat calli and a large evaporative area. *Phylocylapus* differs from *Laetifulvius* in the leaf-like forefemora, the labial segment I reaching the middle of the forecoxa, and tarsal segment I being longer than segments II and III (Poppius 1913). Many characters in the description of the Neotropical genus *Tucuruisca* also fit those of *Laetifulvius*. Both of these genera have an inclined head, wide collar, large hind femur, and tarsal segments subequal in length (Carvalho 1986). *Tucuruisca* differs in the long setae covering the body, the antennal segment I being shorter than the clypeus length and the wide embolium.

*Laetifulvius* is not very similar to any other Australian genus, although it may be related to *Fulviella* and *Phyllofulvius* Carvalho, 1991 as they have a similar structure of the aedeagus with a voluminous endosoma not subdivided into conjunctiva and vesica and bearing numerous sclerites (Fig. 12A, B; Carvalho 1991: figs 4, 11). Both genera differ from *Laetifulvius* in the forefemora being widened. *Fulviella* also differs from *Laetifulvius* in the vertex and the lateral margins of pronotum being carinate, the antennal segment I shorter than the vertex and the tarsal segment I longer than segments II and III each. *Phyllofulvius* differs from *Laetifulvius* in the leaf-shaped antennal segment II (Carvalho 1991).

##### Laetifulvius morganensis sp. nov.

Taxon classificationAnimaliaHeteropteraMiridae

4A9BDA82-2786-5798-89FA-A9BDE14DD310

http://zoobank.org/88354C89-E808-4409-A64F-4CD0E4B6593F

Figs 2 , 11 , 12 , 15B

###### Material examined.

***Holotype***: Australia: South Australia: 51 km NW of Morgan, 33.58333°S, 140°E, 150 m, 01 Nov 1995, Schuh, Cassis, and Gross, 1♂ (UNSW_ENT 00042973) (SAMA). Paratype. AUSTRALIA: South Australia: 51 km NW of Morgan, 33.58333°S, 140°E, 150 m, 01 Nov 1995, Schuh, Cassis, and Gross, 1 sex unknown (00042974) (AM).

###### Diagnosis.

Head reddish brown, antennal segments I and II pale brown; segment II whitish yellow apically; labial segment I reddish brown, segments II and III pale brown to brown; pronotum uniformly brown; scutellum mostly yellow with reddish brown base; pro- and mesopleuron brown; metapleuron whitish yellow with reddish marking dorsally; metathoracic scent gland evaporative area whitish yellow; hemelytron mostly brown, corium with white oval marking posteriorly adjacent to cuneus; cuneus reddish brown; coxae mostly whitish yellow; femora reddish brown with whitish yellow apices; fore- and middle tibiae mostly pale brown; basal part of hind tibia reddish brown, its apical third or half whitish yellow (Fig. 2); endosoma sclerotised at right side, with large irregularly shaped sclerite near apical part of ductus seminis and numerous small sclerites and sclerotised areas (Fig. 12A, B).

###### Description.

**Male.** Body length 3.0–3.1. **Coloration** (Fig. 2). Head reddish brown, with yellow and whitish yellow markings anteriorly; antennal segments I and II pale brown, segment II whitish yellow apically; labial segment I reddish brown, segments II and III pale brown to brown; pronotum uniformly brown; mesoscutum brown with reddish tinge laterally; scutellum mostly yellow, with base and longitudinal stripe reddish brown; pro- and mesopleuron brown; metapleuron whitish yellow with reddish marking dorsally; metathoracic scent gland evaporative area whitish yellow; hemelytron mostly brown, with reddish tinge anteriorly; embolium with reddish tinge posteriorly; corium with white oval marking posteriorly adjacent to cuneus; cuneus reddish brown; membrane pale brown; coxae uniformly whitish yellow or with brown bases; femora reddish brown with whitish yellow apices; fore- and middle tibiae mostly pale brown, reddish brown basally, foretibia sometimes red apically; basal part of hind tibia reddish brown, its apical third or half whitish yellow; tarsi whitish yellow to pale brown. **Surface and vestiture.** As in generic description. **Structure and measurements.** Body ca. 3.0–3.2 × as long as wide, ca. 3.3 × as long as pronotum width; head ca. 2.1–2.2 × as wide as long; vertex ca. 1.1–1.2 × as wide as eye; in anterior view head ca. 1.2–1.3 × as wide as high; antennal segment I ca. 1.1–1.2 × as long as vertex width, ca. 0.4 × as wide as head width; segment II ca. 3.1 × as long as segment I, ca. 3.4–3.7 × as long as vertex width, ca. 1.3 × as long as head width, ca. 0.8 × as long as pronotum width; pronotum ca. 2.0–2.3 × as wide as long, ca. 1.5–1.6 × as wide as head. ***Genitalia*.** Right paramere short, broad, r-shaped, its apical part broad in dorsal view and narrow in posterior view; left paramere ca. 1.5 × as long as right paramere, r-shaped, its apical part twisted in dorsal view and widened in posterior view; basal half of left paramere with wide outgrowth directed inwards (Fig. 12C–F); theca triangular with small tubercles on left hand side (Fig. 12A, B); endosoma not subdivided into vesica and conjunctiva, most part of ductus seminis membranous and coiled, basal part sclerotised with outgrowth directed upwards, apical part sclerotised and widened, subtriangular in dorsal view, with right side elongate; endosoma sclerotised at right side, with large irregularly shaped sclerite near apical part of ductus seminis and numerous small sclerites and sclerotised areas (Fig. 12A, B).

###### Distribution.

Known only from type locality in South Australia (Fig. 15B).

###### Collection techniques.

Both specimens were collected at light.

###### Etymology.

The species is named after town Morgan, as it was collected nearby.

##### Micanitropis gen. nov.

Taxon classificationAnimaliaHeteropteraMiridae

6DF733F1-928B-5D18-B8BB-AB70A4251C97

http://zoobank.org/D54217ED-80D8-49C8-8BC1-4B7D7B9C911B

###### Type species.

*Micanitropis seisia* sp. nov. by original designation.

###### Diagnosis.

**Figure 13.**
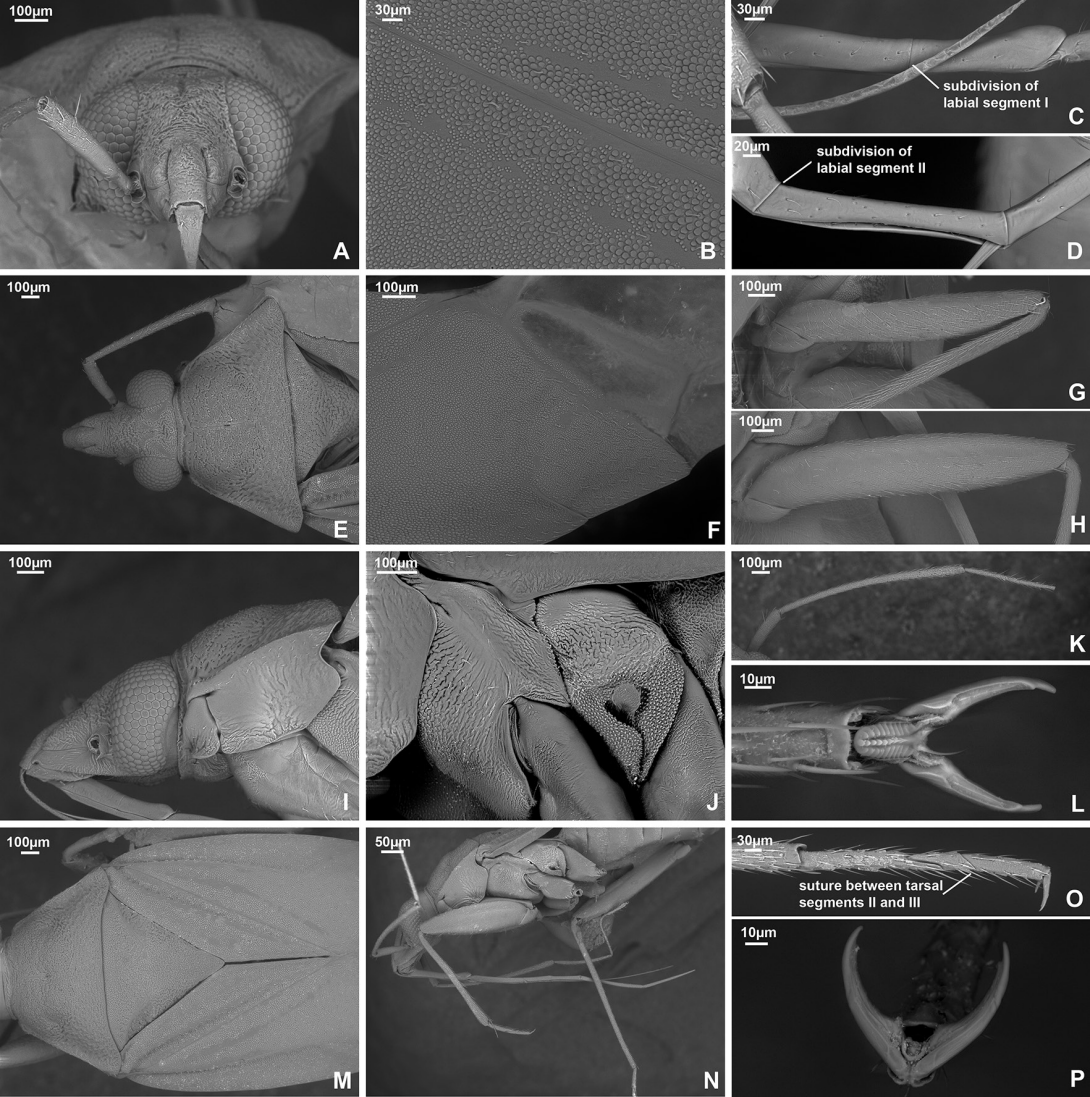
SEM images. *Micanitropis seisia***A** head, anterior view **B** tubercles on hemelytron **C** labial segment I **D** apical part of labial segment II **E** head and pronotum, dorsal view **F** cuneus and membrane cell **G** trichobothria on middle femur **H** trichobothria on hind femur **I** head and pronotum, lateral view **J** pleura **K** antennal segments I, II **L** pretarsus, ventral view **M** scutellum, clavus and corium **N** legs **O** hind tarsus **P** pretarsus, dorsal view.

**Figure 14.**
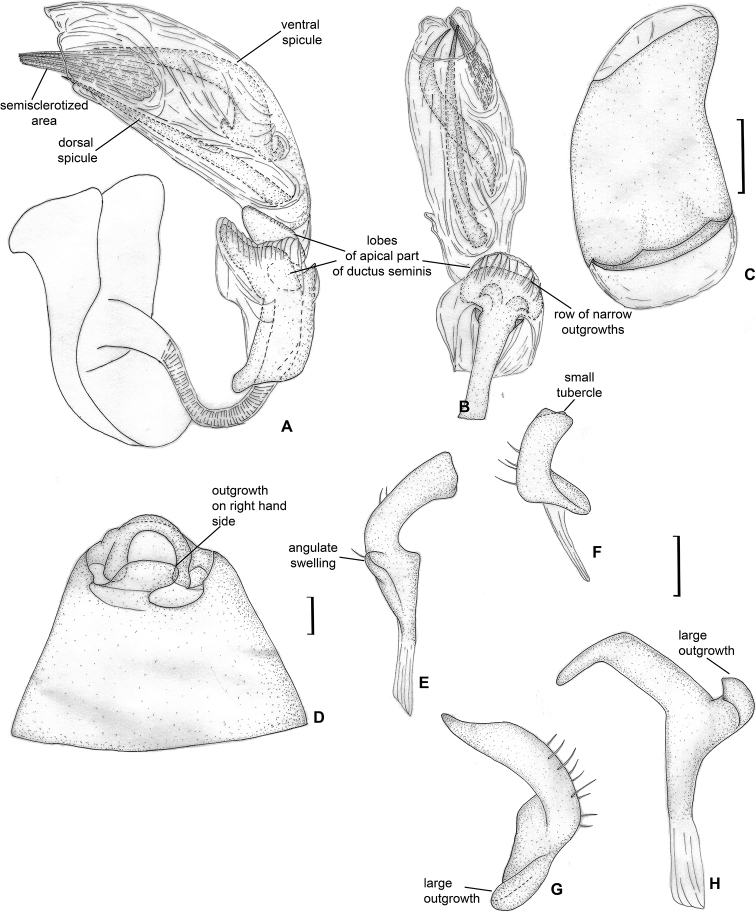
Male genitalia. *Micanitropis seisia***A** aedeagus, left lateral view **B** aedeagus, dorsal view **C** theca **D** genital capsule **E** left paramere, dorsal view **F** left paramere, posterior view **G** right paramere, posterior view **H** right paramere, dorsal view.

*Micanitropis* can be separated from other representatives of Cylapinae using the following combination of characters: body impunctate; dorsum without net-like pattern of microsculpture on head, pronotum and pleura; dorsum clothed with rare short adpressed setae; hemelytron covered with small tubercles and sparse setae; head, pronotum and pleura with distinct rugosities (Fig. 13A, B, E, F, I, J, M); head horizontal, in lateral view longer than high (Fig. 13I); vertex not carinate (Fig. 13E), not raised above eyes (Fig. 13I); length of antennal segment I subequal to vertex width; antennal segment II not modified (Fig. 13K); labium reaching or almost reaching genital segment (Fig. 13N); calli moderately raised, not delimited with depression laterally and posteriorly, delimited from each other with shallow depression; collar narrow, delimited with shallow depression (Fig. 13E); lateral margin of pronotum strongly carinate (Fig. 13I); corium and hind femora without translucent patches; cuneus as long as wide at base (Fig. 13F); hemelytron full, lateral margins rounded, not concave or constricted anteriorly (Fig. 2); metathoracic scent gland evaporative area triangular and large, reaching base of hind coxa (Fig. 13J); forefemur wider than middle and hind femora; tarsal segment I longer than segments II and III each (Fig. 13O); apical part of ductus seminis widened, with two lobes around secondary gonopore both having row of narrow outgrowths along outer margin (Fig. 14A, B).

###### Description.

**Male. Coloration** (Fig. 2). Background colouration brown to dark brown with whitish yellow to pale brown markings and stripes, sometimes with reddish tinge. **Surface and vestiture** (Fig. 13B, E, I, J, M). Body impunctate; scutellum not serrate laterally (Fig. 13M); head, pronotum, scutellum and pleura with distinct rugosities (Fig. 3A, E, I, J, M); hemelytron clothed with small tubercles (Fig. 13B); dorsum and pleura without net-like pattern of microsculpture (Fig. 3A, E, I, J, M). Body clothed with short sparse adpressed setae (Fig. 13B, J); setae on dorsum very short, setae on antenna, pleura, legs and abdomen longer; spines on tibiae short and pale. **Structure.** Body elongate oval. ***Head*.** Horizontal, dorsally as long as or slightly longer than wide (Fig. 13E), vertex not carinate, eye not protruding, not covering anterior margin of pronotum posteriorly (Fig. 13E); in anterior view head wider than high, antennal fossa placed near ventral 1/3 of eye, above ventral margin of eye; base of clypeus not delimited with depression, placed near median of eye (Fig. 13A); in lateral view head longer than high; vertex not raised above eyes; eye large, producing to ventral margin of head; eye located close to lateral margin of pronotum or slightly covering it; antennal fossa slightly removed from eye and placed close to suture between mandibular and maxillary plates; mandibular and maxillary plates not separated from head with suture or depression; labrum triangular, not modified, shorter than labial segment I; buccula elongate, ca. 5–6 × as long as high; distance between buccula and pronotum longer than buccula length (Fig. 13I). ***Antenna*.** Shorter than body, antennal segment I not widened, shorter than head width; antennal segment II slightly widened towards apex, slightly thinner than segment I, longer than head width; segment III slightly shorter than half of segment II; segment IV ca. 1.5 × as long as segment III; segment III as thick as segment IV, and both thinner than segment II (Fig. 13K, N). ***Labium*.** Apex almost reaching or reaching genital segment; labial segment I almost reaching posterior margin of head, subdivided in apical half (Fig. 13C, I, N); segment II almost twice as long as segment I, subdivided subapically, its apical part 9–10 × as long as wide (Fig, 13D, N); segment III subequal to half of segment II, more than 10 × as long as wide; segment IV slightly shorter or same length as segment III (Fig. 13N). ***Thorax*.** Pronotum wider than long (Fig. 13E), lateral margins straight in dorsal view, strongly carinate in lateral view (Fig. 13E, I); collar relatively narrow, narrower than antennal segment I, separated with weak depression; calli large and moderately upraised, covering 2/3 of pronotum surface, separated from each other with weak depression; posterior margin of pronotum slightly concave (Fig. 13E, M); mesoscutum exposed; scutellum flat (Fig. 13M); propleural apodeme T-shaped (Fig. 13I); mesopleural apodeme slit-like; mesothoracic spiracle slit-like, with row of microsculpture along anterior margin; metathoracic gland evaporative area triangular, lateral margin reaching base of hind coxa; peritreme upraised, rounded; metepimeron narrow (Fig. 13J). ***Hemelytron*** (Fig. 13F, M). Outer margins rounded, not concave or constricted anteriorly; clavus with longitudinal ridge; claval commissure slightly longer than scutellum; medial fracture almost reaching middle of corium; ridge along medial fracture distinct basally; embolium relatively wide, its widest part subequal to 1/3 of cuneus width; R+M distinct only basally; cuneus visibly delimited, as wide as long, not incised at base; membrane with two cells, distance between cell and membrane apex slightly longer than cell length. ***Legs*.** Forecoxa slightly shorter than pronotum length, longer and wider than middle and hind coxae; forefemur widened, 3–4 × as long as wide, wider and slightly longer than middle femur, slightly wider and shorter than hind femur (Fig. 13G, H, N), segment I of hind tarsus twice longer than segment II; segment III slightly longer than segment II (Fig. 13O); claw with subapical tooth; middle row of tiles on unguitractor full (Fig. 13L). ***Genitalia*.** See species description.

**Female.** Similar to male. ***Genitalia*.** See species description.

###### Etymology.

The genus is named for its sparkling appearance, *micans* from Latin meaning sparkling, glittering. The genus is masculine.

###### Remarks.

*Micanitropis* belongs to Fulviini as its structure fits the diagnosis for this tribe, in particular, it has the prognathous head, the antenna shorter than the body, the body impunctate, and its labium is long, reaching or almost reaching the genitalia segment (Gorczyca 2000).

*Micanitropis* is similar to *Peritropis* Uhler, 1891, as they both have the moderately elevated calli, the carinate lateral margins of pronotum, the eyes elongate in lateral view and reaching gula, and the hemelytron, at least in some species, including type species *P. saldaeformis* Uhler, 1891, is covered with small tubercles (Moulds and Cassis 2006; Wolski and Henry 2012). Currently *Peritropis* includes ca. 90 species worldwide, with only the Australian and American fauna having been revised (Moulds and Cassis 2006; Wolski and Henry 2012). According to the previous works and personal observations, *Peritropis* differs from *Micanitropis* in the collar being indistinct, the vertex usually more or less carinate, the metathoracic scent gland evaporative area usually being reduced or at least its anterior angle being rounded, and the head, pronotum and pleura not being covered with rugosities, but instead with a net-like pattern of microsculpture. The anterior margin of pronotum of *Peritropis* is also often concave and angulate at sides, and the apical part of ductus seminis does not have the row of outgrowths along margin (Moulds and Cassis 2006; Wolski and Henry 2012).

As mentioned in the remarks for *Callitropisca*, this genus, *Micanitropis*, and *Xenocylapidius* share similar genitalic structures, with the apical part of the ductus seminis having two lobes, bearing a row of long outgrowths along outer margin. See comparison of *Callitropisca* and Micanitropis in the Remarks section for Callitropisca. *Xenocylapidius* and *Micanitropis* are similar in the head being longer than high in lateral view, the vertex flat (Fig. 13I; Wolski and Gorczyca 2014b: figs 9–15), and the tarsal segment I being longer than tarsal segments II and III each (Fig. 13O). However, *Xenocylapidius* differs from *Micanitropis* in the antennal segment I being longer than the vertex, the setae covering the dorsum are either dense and short or long and sparse, and the head, pronotum, and scutellum are not rugose or only slightly rugose (pers. obs.; Wolski and Gorczyca 2014b).

##### Micanitropis seisia sp. nov.

Taxon classificationAnimaliaHeteropteraMiridae

C16BC8B2-924A-5AE3-A52D-ACCD0EFF1A21

http://zoobank.org/B2AE0B02-5998-4EA5-9237-2540B9C2747E

**Figure 15.**
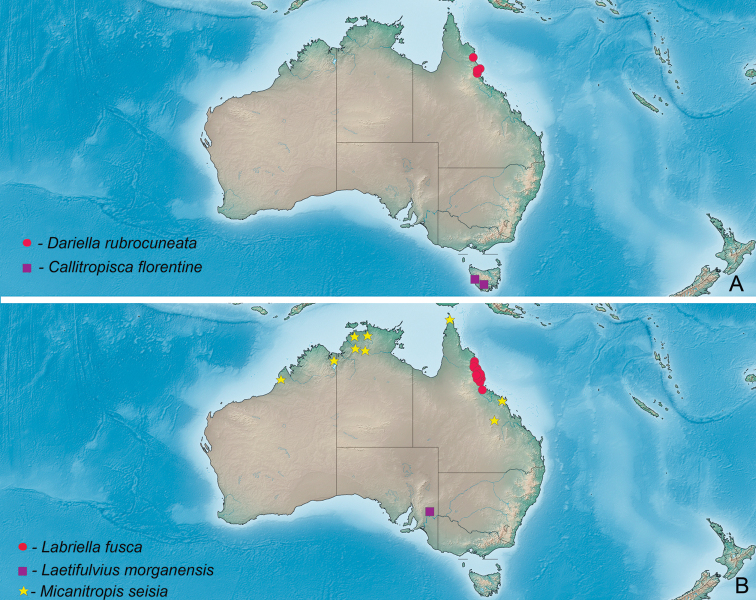
Distribution maps **A***Dariella rubrocuneata*, *Callitropisca florentine***B***Labriella fusca*, *Laetifulvius morganensis*, *Micanitropis seisia*.

Figs 2 , 13 , 14 , 15B

###### Material examined.

***Holotype***: Australia: Queensland: Seisia via Bamaga, 10.85283°S, 142.37132°E, 10 Jan 2011, J. Sailor, 1♂ (UNSW_ENT 00027641) (QM). ***Paratypes***: Australia: Northern Territory: Crocodile Ck nr Dorisvale M.T., 14.29°S, 131.22°E, 17 Nov 1984, M. B. Malipatil, 1♂ (00043059) (NTM). Kakadu National Park, Nourlangie Camp, 12.759°S, 132.659°E, 17 Nov 1979–18 Nov 1979, M. B. Malipatil, 1♀ (00043063) (NTM). Lake Bennett, 19 km SE off Stuart Hwy nr Manton Dam, 12.86449°S, 131.11889°E, 30 Mar 1979, M. B. Malipatil, 3♂ (00043052–00043054), 1♀ (00043055) (NTM), 1 sex unknown (00043056) (NTM). Tindal, 14.516°S, 132.383°E, 01 Dec 1967–20 Dec 1967, W. Vestjens, 1♂ (00043060) (NTM). Queensland: Clermont, 22.823°S, 147.638°E, Nov 1929, K. K. Spence, 1♂ (00043061) (AM). Proserpine, Thompson Creek, (site XY15), 20.51888°S, 148.55694°E, 21 m, 11 Nov 2007, C. J. Burwell, 1♂ (00043046) (QM). Proserpine, Thompson Creek, site XY15, 20.519°S, 148.557°E, 30 m, 11 Nov 2007, C. J. Burwell, 2♀ (00043048, 00043049) (QM). Western Australia: Kimberley Research Station, via Kununura, 15.70777°S, 128.69947°E, 25 Nov–26 Nov 1997, A. Postle and C. Brockway, 1♀ (00043062) (WAM). Roebuck Plains, via Broome, 17.96°S, 122.435°E, 30 Dec 1997–02 Jan 1998, C. Johnstone, 1♂ (00043057) (WAM).

###### Diagnosis.

Head mostly brown to dark brown dorsally; antennal segment I yellow basally and reddish apically; segment II pale brown to brown, whitish yellow apically; segments III and IV pale brown to brown; pronotum, mesoscutum and scutellum brown to dark brown; scutellum whitish yellow to yellow apically; pleura mostly brown to dark brown or reddish brown; hemelytron mostly brown; clavus with three longitudinal whitish yellow stripes, inner stripe sometimes faint or indistinct; corium with two longitudinal whitish yellow stripes reaching or almost reaching middle of corium (Fig. 2); vesica with two elongate sclerites, one of them straight and placed dorsally, second one curved and widened basally, vesica also with triangular semi-sclerotised area apically (Fig. 14A, B).

###### Description.

**Male.** Body length 3.1–3.4. **Coloration** (Fig. 2). Background colouration brown to dark brown. ***Head*.** Brown to dark brown dorsally with yellow marking near inner margin of eye; mandibular and maxillary plates and ventral side of head pale brown often with reddish tinge; antennal segment I yellow basally and reddish apically; segment II pale brown to brown, whitish yellow apically; segments III–IV pale brown to brown; labium yellow to brown, labial segment I sometimes with reddish tinge. ***Thorax*.** Pronotum uniformly brown to dark brown. Mesoscutum and scutellum brown to dark brown; mesoscutum often with pale brown or reddish marking laterally; scutellum whitish yellow to yellow apically; pleura brown to dark brown, sometimes reddish brown; evaporative area often slightly paler than metapleuron. ***Hemelytron*.** Mostly brown; clavus with three longitudinal whitish yellow stripes, inner stripe sometimes faint or indistinct; corium with two longitudinal whitish yellow stripes reaching or almost reaching middle of corium; embolium whitish yellow anteriorly; corium and embolium with yellow marking adjacent to cuneus, sometimes with reddish tinge; membrane pale brown with brown cells. ***Legs*.** Forecoxa brown to dark brown, often with whitish apex; middle and hind coxae whitish yellow to pale brown; femora brown, yellow or reddish yellow apically; forefemur often darker than middle and hind femora; tibiae pale brown to dark brown, often yellow apically; tarsi whitish yellow to pale brown. ***Abdomen*.** Brown to dark brown, sometimes with reddish tinge. **Surface and vestiture.** See generic description. **Structure and measurements.** Body ca. 2.5–2.8 × as long as wide, ca. 2.7–3.1 × as long as pronotum width; head ca. 1.0–1.3 × as wide as long, in lateral view head ca. 1.4–1.5 × as long as high; antennal segment I ca. 1.3–1.6 × as long as vertex, ca. 0.5–0.6 × as long as head width; antennal segment II ca. 2.8–3.0 × as long as segment I, ca. 4.0–4.3 × as long as vertex width, ca. 1.6–1.7 × as long as head width, ca. 0.8–0.9 × as long as pronotum width; pronotum ca. 1.8–2.0 × as wide as head, ca. 1.9–2.2 × as wide as long. ***Genitalia*.** Genital capsule as long as wide, with outgrowth on right hand side on posterior margin dorsally (Fig. 14D). Parameres r-shaped, subequal in length; basal part of right paramere with angulate swelling directed inwards and rounded outgrowth directed upwards, apical part of right paramere widened with small tubercle apically; left paramere with large outgrowth on basal part directed outwards and upwards, apical process narrow and elongate (Fig. 14E–H); theca without outgrowths (Fig. 4C); endosoma subdivided into vesica and conjunctiva; vesica with two elongate spicules, tapering apically, dorsal spicule straight, not particularly widened basally; ventral spicule curved, and widened basally in lateral view; vesica also with triangular semi-sclerotised area apically; ductus seminis widened and sclerotised apically; secondary gonopore surrounded with two wide lobes, with row of narrow outgrowths along outer margin, one of those lobes placed dorsally and another one ventrally (Fig. 14A, B).

**Female.** Body length 3.1–3.7. **Coloration** (Fig. 2). As in male, marking near eye sometimes absent, cuneus sometimes almost entirely whitish yellow. **Structure and measurements.** Body ca. 2.2–2.7 × as long as wide, ca. 2.7–3.3 as long as pronotum width; head ca. 0.9–1.0 × as wide as long, in lateral view head ca. 1.5 × as long as high; antennal segment I ca. 1.2–1.6 × as long as vertex, ca. 0.5–0.7 × as long as head width; antennal segment II ca. 3.1–3.2 × as long as segment I, ca. 3.6–4.9 × as long as vertex width; ca. 1.4–2.0 × as long as head width, ca. 0.8–0.9 × as long as pronotum width; pronotum ca. 1.8–2.3 × as wide as head, ca. 2.0–2.2 × as wide as long. ***Genitalia*.** Dorsal labiate plate as long as wide, with large elongate sclerotised rings, each of them ca. 0.2 × as wide as dorsal labiate plate, and only slightly shorter than dorsal labiate plate; sclerotised rings connected with each other with transversal sclerite anteriorly; posterior part of dorsal labiate plate with paired sclerotised areas and paired membranous areas with small tubercles (Fig. 10B); ventral wall with v-shaped medial sclerite surrounding vulva (Fig. 10D).

###### Distribution.

Known from different locations in the dry areas in the northern parts of Western Australia, Northern Territory and Queensland (Australia) (Fig. 15B).

###### Collection techniques.

The specimens were collected with hands at night, at light, at MV light, and using pyrethrum knockdown of mango trees.

###### Etymology.

Named after the town Seisia in the Cape York (Queensland, Australia), where the holotype was collected (Fig. 15B).

## Discussion

The subfamily Cylapinae is characterised by long and narrow tarsi, as well as the combination of characters in the pretarsus, specifically the setiform and often asymmetrical parempodia, the lack of pulvilli, the slender claws usually toothed apically, and the three rows of tiles on the unguitractor with the tiles of the medial row acute (Schuh 1976; Gorczyca 2000; Namyatova et al. 2016). The listed characters occur in Bothriomirini, Cylapini, Fulviini and Rhinomirini (Wolski et al. 2016; Namyatova and Cassis 2016a; Wolski 2017; Namyatova et al. 2016, 2019). However, Vanniini has a different structure of the pretarsus with flattened spatulate parempodia and without a middle row of tiles on the unguitractor plate (Namyatova et al. 2016). A combination of characters similar to that possessed by the most cylapines was found in Isometopinae and Psallopinae. Isometopinae are different from Cylapinae in the presence of ocelli (Namyatova and Cassis 2016b), and Psallopinae are often very small (1–2 mm), fragile and have the vertex width shorter than eye diameter (Namyatova and Cassis 2019b). All genera described in this paper have the abovementioned structures of the pretarsus and do not fit the diagnoses of Isometopinae and Psallopinae, and, therefore, we place them into Cylapinae.

The current diagnoses for Cylapini and Fulviini cannot be applied to all representatives of those tribes, and this complicates the tribal assignment of the new genera. *Callitropisca* and *Micanitropis* possess all the features typical for Fulviini. Although *Laetifulvius* has the head strongly inclined, its morphological features also fit the current understanding of Fulviini in all other respects.

*Labriella* and *Dariella* do not fit the diagnoses for any of the Cylapinae tribes. They have a vertical head, which is true for Cylapini, Bothriomirini and Vanniini. However, according to the diagnoses, the Cylapini and Vanniini typically have antennae longer than the body, whereas they are shorter than the body in *Labriella* and *Dariella*. The combination of the vertical head and short antennae is characteristic for Bothriomirini. All representatives of Bothriomirini form a well-defined group with some important characters not occurring in *Dariella* and *Labriella* (see Remarks section for those genera). However, Cylapini have other genera which also have short antennae, and those taxa are similar to *Dariella* and *Labriella*. In particular, *Dariella* has the male genitalia and pretarsus structure very similar to another Australian genus *Carvalhoma*, and the metathoracic scent gland evaporative area similar to *Schizopteromiris*, which is also known from Australia. Therefore, we place *Dariella* and *Labriella* in Cylapini based on their affinities to other genera assigned to this tribe, although not its typical representatives. We are awaiting the phylogenetic study of Cylapinae to clarify the position of those two new genera.

With five new monotypic genera, Australian Cylapinae fauna now includes 26 genera and 48 species currently assigned to four tribes, Bothriomirini, Cylapini, Fulviini and Vanniini. The Cylapini fauna is composed of the genera *Carvalhoma*, *Dariella*, *Labriella* and *Schizopteromiris*. None of these genera fully fit the diagnosis of this tribe, and the position of those genera can be reassessed in the future.

## Supplementary Material

XML Treatment for Dariella

XML Treatment for Dariella rubrocuneata

XML Treatment for Labriella

XML Treatment for Labriella fusca

XML Treatment for Callitropisca

XML Treatment for Callitropisca florentine

XML Treatment for Laetifulvius

XML Treatment for Laetifulvius morganensis

XML Treatment for Micanitropis

XML Treatment for Micanitropis seisia
